# FKBP4 integrates FKBP4/Hsp90/IKK with FKBP4/Hsp70/RelA complex to promote lung adenocarcinoma progression via IKK/NF-κB signaling

**DOI:** 10.1038/s41419-021-03857-8

**Published:** 2021-06-10

**Authors:** Shuai Zong, Yulian Jiao, Xin Liu, Wenli Mu, Xiaotian Yuan, Yunyun Qu, Yu Xia, Shuang Liu, Huanxin Sun, Laicheng Wang, Bin Cui, Xiaowen Liu, Ping Li, Yueran Zhao

**Affiliations:** 1 Department of Central Lab, Shandong Provincial Hospital, Cheeloo College of Medicine, Shandong University, Jinan, China; 2 The Second Affiliated Hospital of Shandong University of Traditional Chinese Medicine, Jinan, China; 3 Department of Clinical Laboratory, Qilu Hospital, Shandong University, Jinan, China; 4 Department of Thoracic Surgery, Shandong Provincial Hospital, Cheeloo College of Medicine, Shandong University, Jinan, China; 5 Center for Reproductive Medicine, Shandong University, National Research Center for Assisted Reproductive Technology and Reproductive Genetics, the Key Laboratory for Reproductive Endocrinology of Ministry of Education, Jinan, China

**Keywords:** Non-small-cell lung cancer, Cell signalling

## Abstract

FKBP4 belongs to the family of immunophilins, which serve as a regulator for steroid receptor activity. Thus, FKBP4 has been recognized to play a critical role in several hormone-dependent cancers, including breast and prostate cancer. However, there is still no research to address the role of FKBP4 on lung adenocarcinoma (LUAD) progression. We found that FKBP4 expression was elevated in LUAD samples and predicted significantly shorter overall survival based on TCGA and our cohort of LUAD patients. Furthermore, FKBP4 robustly increased the proliferation, metastasis, and invasion of LUAD in vitro and vivo. Mechanistic studies revealed the interaction between FKBP4 and IKK kinase complex. We found that FKBP4 potentiated IKK kinase activity by interacting with Hsp90 and IKK subunits and promoting Hsp90/IKK association. Also, FKBP4 promotes the binding of IKKγ to IKKβ, which supported the facilitation role in IKK complex assembly. We further identified that FKBP4 TPR domains are essential for FKBP4/IKK interaction since its association with Hsp90 is required. In addition, FKBP4 PPIase domains are involved in FKBP4/IKKγ interaction. Interestingly, the association between FKBP4 and Hsp70/RelA favors the transport of RelA toward the nucleus. Collectively, FKBP4 integrates FKBP4/Hsp90/IKK with FKBP4/Hsp70/RelA complex to potentiate the transcriptional activity and nuclear translocation of NF-κB, thereby promoting LUAD progression. Our findings suggest that FKBP4 may function as a prognostic biomarker of LUAD and provide a newly mechanistic insight into modulating IKK/NF-κB signaling.

## Introduction

Lung cancer is the most common cause of cancer-related mortality worldwide^1,2^. Approximately 85% of diagnosed cases are classified as non-small-cell lung cancer (NSCLC), with lung adenocarcinoma (LUAD) being the predominant histological subtype^3^. Despite recent advances in treatments such as platinum therapy and radiotherapy, the clinical outcomes of LUAD patients remain unsatisfactory due to late diagnosis and high rates of metastasis^4^. Therefore, deciphering gene alterations and mechanisms underlying the initiation and progression of LUAD is beneficial to discovering novel diagnostic biomarkers and potential therapeutic targets.

Activation of nuclear factor-κB (NF-κB) signaling has been shown to contribute to tumorigenesis and development in various cancers^5–8^. In resting cells, NF-κB heterodimers reside in the cytoplasm due to the interaction with the inhibitory factor IκB. The canonical pathway is normally triggered by proinflammatory cytokines (e.g., tumor necrosis factor-α (TNF-α) and interleukin-1 (IL-1)), pathogen-associated molecules (e.g., lipopolysaccharide (LPS)), and chemical inducers (e.g., phorbol-12-myristate-13-acetate (PMA)) that activate the tripartite IκB kinase complex (IKK; IKKα/IKKβ/IKKγ). Under certain stimuli, activated IKKβ, which confers Ser/Thr kinase activity, directly phosphorylates IκBα at Ser32/36 and induces its degradation via the 26S proteasome. Subsequently, the liberated NF-κB heterodimers, consisting predominantly of RelA (p65) and NF-κB1 (p50) subunits, translocate into the nucleus and mediate the transcription of target genes.

FK506-binding proteins (FKBPs) belong to the family of immunophilins, which are structurally characterized by the existence of peptidyl-prolyl isomerase (PPIase) domains^9^. In addition to their well-established role in immunosuppression, FKBPs are involved in numerous cellular processes, such as protein trafficking, transcriptional regulation, protein folding, and signal transduction^10^. The PPIase domains are located at the N-terminal end of FKBPs and consist of FKBP12-like domains 1 and 2 (FK1 and FK2). Only the FK1 domain confers PPIase enzymatic activity that can be inhibited by the immunosuppressants FK506 and rapamycin, while the FK2 domain seems to play a structural role. High-molecular-weight immunophilins, such as FKBP4 and FKBP5, possess C-terminal tetratricopeptide repeat (TPR) domains, which are able to form complexes with the molecular chaperone Hsp90^11^, and participate in protein−protein interactions^12^.

As a co-chaperone, FKBP4 modulates the activity of steroid receptors (SRs), including glucocorticoid (GR), mineralocorticoid (MR), androgen (AR), progesterone receptors (PR), as well as estrogen receptor (ER)^13–16^. This is particularly important for the development of hormone-dependent breast cancer^17,18^ and prostate cancer^19,20^. The upregulation of FKBP4 was also detected in hepatocellular carcinoma (HCC), which was strongly related to HCC staging^21^. Notably, a recent study reported that FKBP4 potentiated AKT signaling to facilitate the proliferation and survival of triple-negative breast cancer cells, suggesting that the regulatory mechanism is independent of ER, at least in these ER-negative models^22^. Additionally, FKBP4 regulates the activity of other client proteins such as Tau, the hallmark of several human neurodegenerative diseases^12,23^. In this study, we characterized the biological role and regulatory mechanism of FKBP4 overexpression in LUAD progression.

## Results

### FKBP4 is highly expressed in LUAD and correlated with poor prognosis

**Fig. 1:**
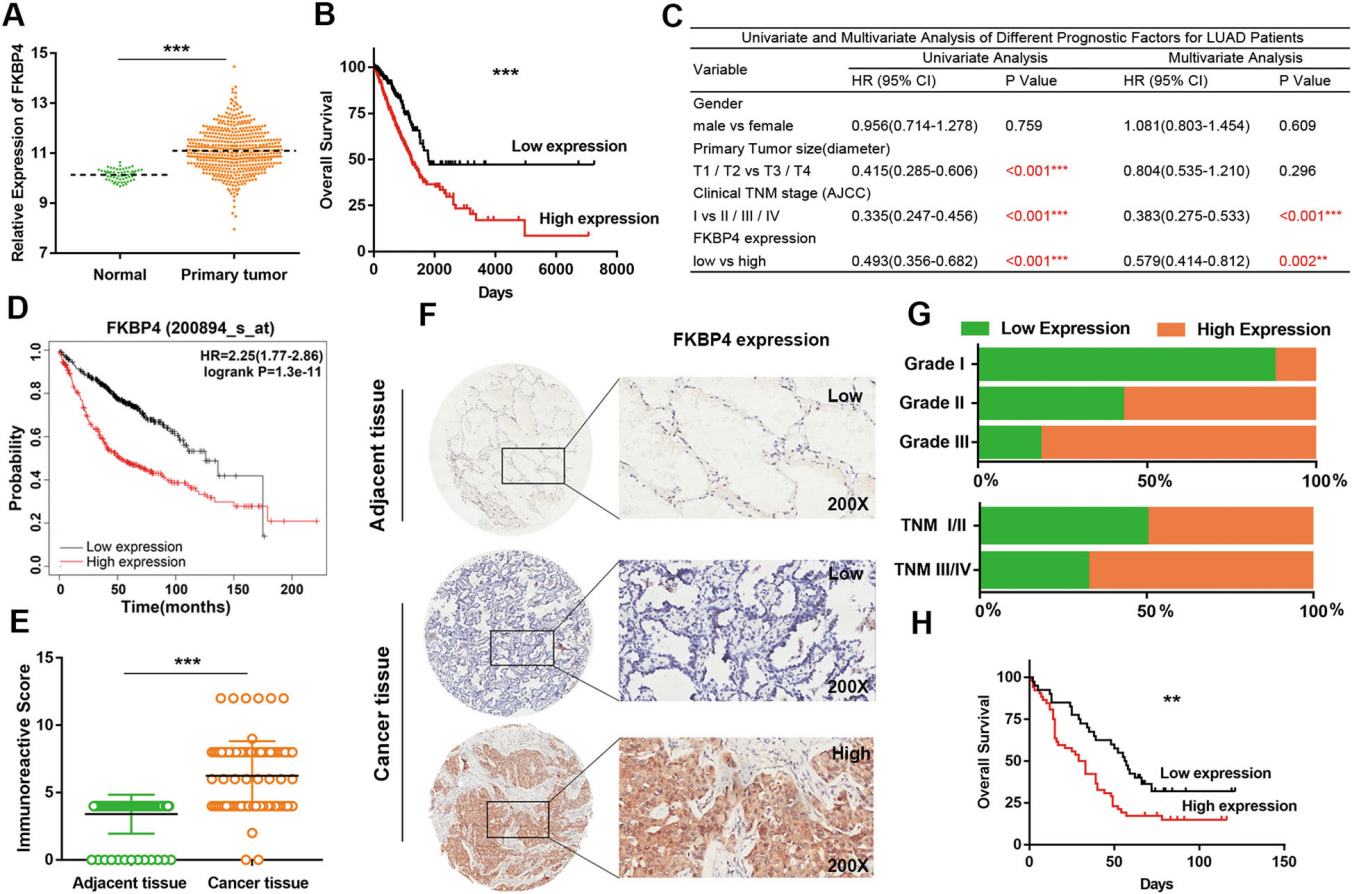
FKBP4 is overexpressed and predictive of poor prognosis for LUAD patients. **A** The mRNA levels of FKBP4 in human LUAD (*n* = 515) were remarkably higher than those in normal tissues (*n* = 59) based on TCGA database. **B** Kaplan−Meier analysis showed that LUAD patients with high FKBP4 expression had a shortened overall survival (OS) (cut-off value = 10.83). **C** Uni- and multivariate Cox model analysis illustrating the prognostic significance of FKBP4 expression in LUAD. **D** The Kaplan−Meier plotter online database verified that FKBP4 overexpression was associated with a poor OS (*n* = 719). **E** The protein levels of FKBP4 in LUAD (*n* = 94) were increased compared with those in adjacent normal tissues (*n* = 86) as determined by IHC analysis. **F** Representative IHC staining images of FKBP4 in adjacent and LUAD tissues. **G** Quantification analysis of FKBP4 staining in LUAD with different grades and with different clinical stages. (**H**) Kaplan−Meier analysis confirmed the prognostic value of FKBP4 protein levels in LUAD. (**P < 0.01, ***P < 0.001).

To explore the potential role of FKBP4 in LUAD progression, we analyzed the RNA-seq data (FPKM values) from The Cancer Genome Atlas (TCGA) database. The results showed that FKBP4 expression was significantly increased in LUAD tissues (*n* = 515) compared with normal tissues (*n* = 59, absolute fold change = 1.92, *P* < 0.001) (Fig. 1A). Similar results were obtained with three other independent datasets from Oncomine database (Supplementary Fig. S1A). Moreover, we found that the FKBP4 expression levels (best cut-off value = 10.8319, Supplementary Fig. S1B) were notably associated with gender, tumor size, and clinical TNM stage according to TCGA database (Table 1). Kaplan−Meier curves showed that patients with high FKBP4 expression had a worse overall survival (OS) than those with low FKBP4 expression (Supplementary Table S1 and Fig. 1B). As shown in Fig. 1C, the univariate Cox model revealed that FKBP4 expression, primary tumor size, and clinical TNM stage were significantly associated with a shorter OS in LUAD patients. Multivariate Cox model analysis showed that FKBP4 expression may play an independent role in LUAD, along with the TNM stage. We next used the Kaplan−Meier plotter online database to examine the association between FKBP4 overexpression and survival of LUAD patients (*n* = 719). The results verified that FKBP4 overexpression was associated with a poor OS (Fig. 1D).

**Table 1:** Correlation between expression of FKBP4 and clinicopathological characteristics of LUAD patients.

Variable	Case	Expression of FKBP4		Chi-square test	
		Low	High	χ^2^	*P*
Median age					
≤66 years	251	94	157	0.106	0.745
>66 years	247	96	151		
Gender					
Male	240	75	165	9.416	0.002**
Female	277	123	154		
Primary tumor size (diameter)					
T1−T2	448	183	265	10.909	0.001**
T3−T4	66	13	53		
Lymph node metastasis					
N−	333	135	198	3.544	0.06
N+	172	55	117		
Distant metastasis					
M0	347	129	218	1.751	0.186
M1	25	6	19		
Clinical TNM stage (AJCC)					
I	277	123	154	8.926	0.003**
II/III/IV	232	73	159		
EGFR genetic status					
Wild-type	197	75	122	0.021	0.885
Mutants	33	13	20		

**P < 0.01

Furthermore, the tissue microarray (TMA) was employed to detect FKBP4 protein expression in patients with LUAD. The percentage of samples with high FKBP4 expression (56.4%, 53/94) was significantly higher in LUAD specimens than in adjacent normal tissues (0%, 0/86) (Fig. 1E, F). We assessed the association between FKBP4 protein levels and different clinicopathologic variables, revealing that FKBP4 expression was positively correlated with the pathological grade and TNM stage (Table 2). In detail, the percentage of high FKBP4 expression was significantly higher in grade II and grade III than in grade I LUAD samples. The percentage of samples with high FKBP4 expression was higher in TNM stages III/IV than TNM stages I/II LUAD samples (Fig. 1G). Additionally, Kaplan−Meier analysis further confirmed that FKBP4 might serve as a biomarker of poor prognosis for LUAD patients (Fig. 1H).

**Table 2:** Correlation between expression of FKBP4 and clinicopathological characteristics of LUAD patients.

Variable	Case	Expression of FKBP4		Chi-square test	
		Low	High	χ^2^	*P*
Median age					
≤61 years	47	20	27	0.043	0.835
>61 years	47	21	26		
Gender					
Male	51	21	30	0.27	0.603
Female	43	20	23		
Grade					
I	8	7	1	6.847	0.009**
II/III	86	34	52		
Primary tumor size (diameter)					
T1−T2	71	32	39	0.249	0.618
T3−T4	23	9	14		
Lymph node metastasis					
N−	39	20	19	0.882	0.348
N+	37	15	22		
Clinical TNM stage (AJCC)					
I/II	45	25	20	5.092	0.024*
III/IV	44	14	30		
EGFR (FISH)					
Negative	73	31	42	0.271	0.603
Positive	14	7	7		

*P < 0.01, **P < 0.01

### FKBP4 potentiates NF-κB pathway and transcriptional activity through IKKβ

**Fig. 2:**
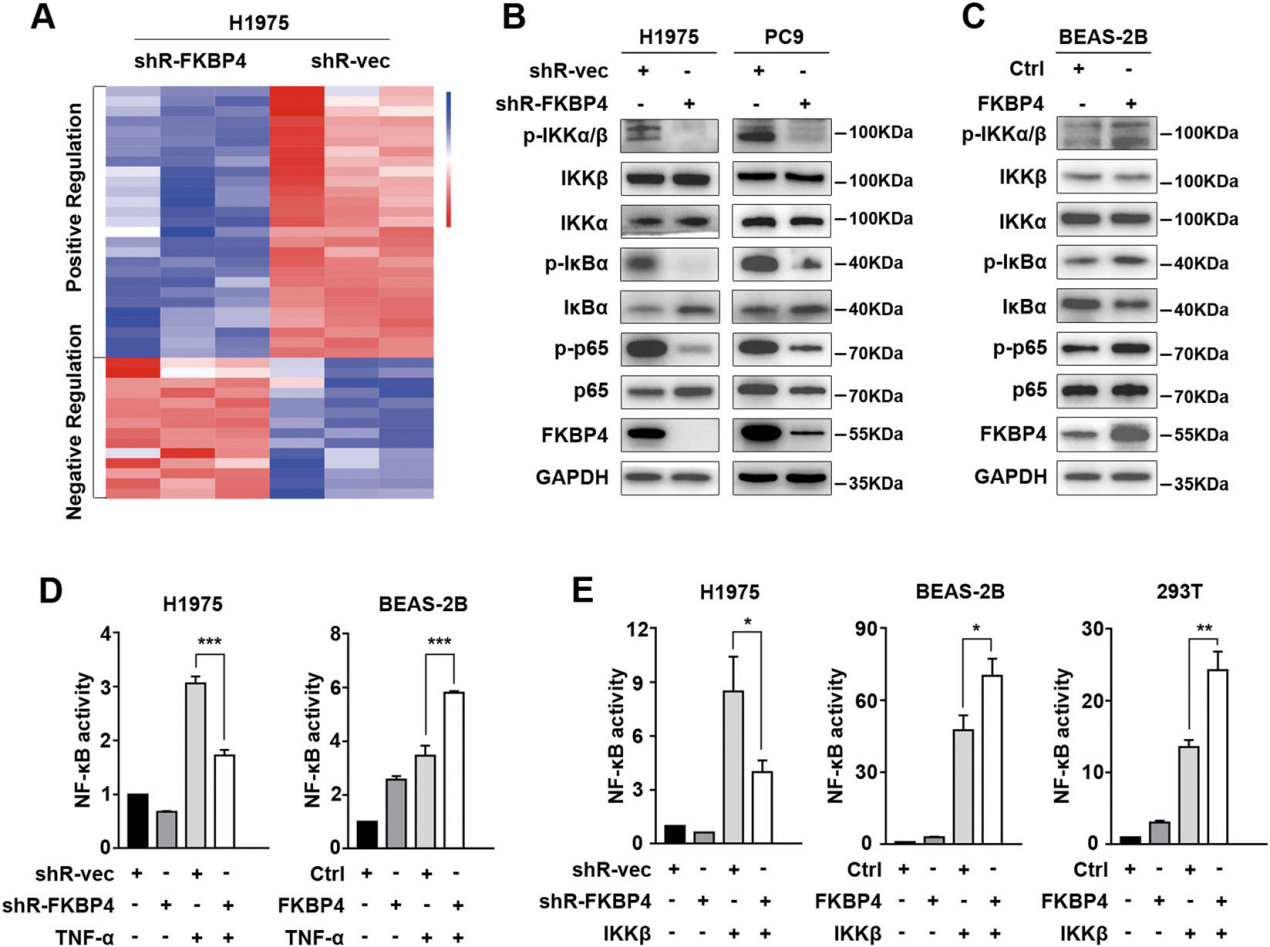
FKBP4 activates NF-κB through IKKβ. **A** Heat map of the normalized relative abundance levels of IKK/NF-κB pathway-related genes significantly changed between the control and FKBP4-knockdown groups. These key genes positively (or negatively) regulated IKK/NF-κB signaling and NF-κB transcriptional activity decreased (or increased) after FKBP4 inhibition. **B** Western blot analysis of IKK/NF-κB signaling molecules in PC9 and H1975 cells transfected with FKBP4 shRNA. **C** Western blot analysis of IKK/NF-κB signaling molecules in BEAS-2B cells overexpressing FKBP4. **D** Inhibition of FKBP4 attenuated TNF-α-induced NF-κB activity in H1975 cells, while overexpression of FKBP4 promoted TNF-α-induced NF-κB activity in BEAS-2B cells. These cells were stimulated with 10 ng/ml TNF-α for 6 h. The transcriptional activity of NF-κB was measured with a luciferase reporter and normalized to Renilla activity. **E** FKBP4 promotes NF-κB transcriptional activity through IKKβ. Luciferase reporter assays of H1975, BEAS-2B, and 293T cells cotransfected with FKBP4 shRNA (or FKBP4) and IKKβ plasmids were performed as indicated (*P < 0.01, **P < 0.01, ***P < 0.001).

Given the putative role of FKBP4 in LUAD, we established the mRNA expression profiles in H1975 cells after knockdown of FKBP4 with shRNA. As shown in Fig. 2A, the RNA sequencing data revealed that FKBP4 downregulation inhibited the IKK/NF-κB pathway and transcriptional activity (Supplementary Table S2). Consistent with the results of RNA sequencing data, depletion of FKBP4 obviously decreased the phosphorylation of IKKα/β, IκBα, as well as RelA without affecting their total protein levels in PC9 and H1975 cell line models (Fig. 2B). In contrast, the opposite results were observed in FKBP4-overexpressing BEAS-2B cells, which are normal lung epithelial cells (Fig. 2C). Dual-luciferase reporter assays were used to assess the effects of differential FKBP4 expressions on NF-κB transcriptional activity. The results showed that knockdown of FKBP4 potently inhibited TNF-α-induced NF-κB activity in H1975 cells, while overexpression of FKBP4 enhanced TNF-α-induced NF-κB activity in BEAS-2B cells (Fig. 2D).

Due to the impact of FKBP4 on IKKβ phosphorylation, we determined whether FKBP4 enhanced NF-κB activity through IKKβ. Luciferase reporter assays showed that FKBP4 depletion significantly decreased IKKβ-activated NF-κB in H1975, whereas overexpression of FKBP4 increased IKKβ-activated NF-κB in BEAS-2B and 293T cells (Fig. 2E). These results suggest that FKBP4 regulates NF-κB signaling through IKKβ.

### FKBP4 promotes Hsp90/IKK association and IKK complex assembly

**Fig. 3:**
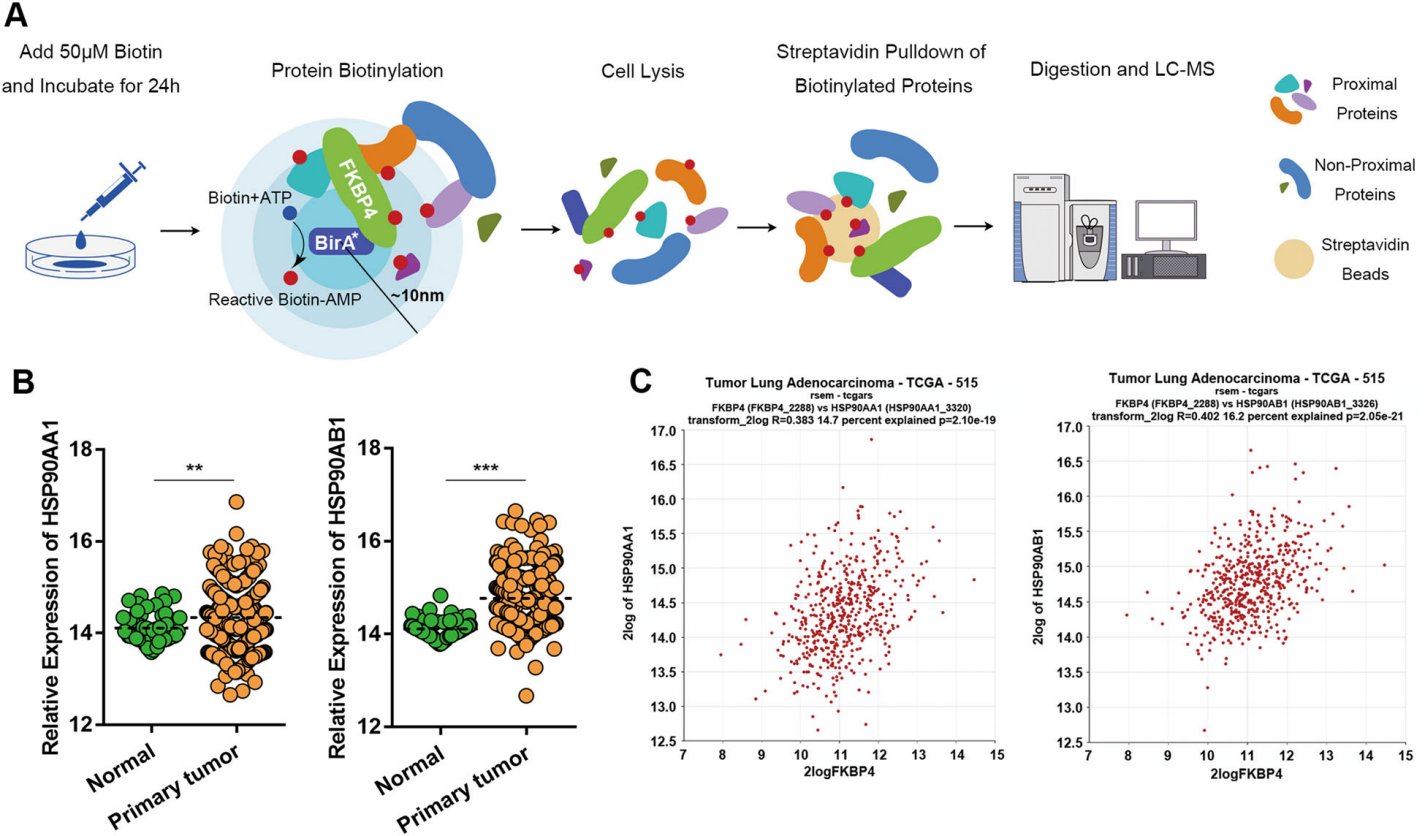
FKBP4 interacts with Hsp90 in LUAD. **A** Schematic representation of the proximity-dependent biotin labeling and identification of proteins associated with HA-BirA*-FKBP4. **B** The mRNA expression levels of HSP90AA1 and HSP90AB1 in LUAD (*n* = 515) were remarkably increased compared with those in normal tissues (*n* = 59) using TCGA database. **C** Correlation of FKBP4 and Hsp90 mRNA expression in LUAD samples from the R2 online database.

To decipher the molecular mechanisms of FKBP4 in NF-κB regulation, we investigated the FKBP4 interactome through the BioID technique according to a previously described protocol (Fig. 3A and Supplementary Table S3)^24,25^. Among these proximal proteins, we focused on Hsp90 as an additional component of the IKK complex^26^, which is required for IKK phosphorylation induced by TNF-α^27–29^.

Next, we examined the mRNA expression of Hsp90, including HSP90AA1 and HSP90AB1 isoforms, in LUAD patients using TCGA database. The results showed that Hsp90 expression was elevated in tumor tissues (*n* = 515) compared with normal tissues (*n* = 59) (Fig. 3B). Furthermore, correlation analysis verified the positive association between FKBP4 and Hsp90 expression in LUAD (R2 online database) (Fig. 3C).

**Fig. 4:**
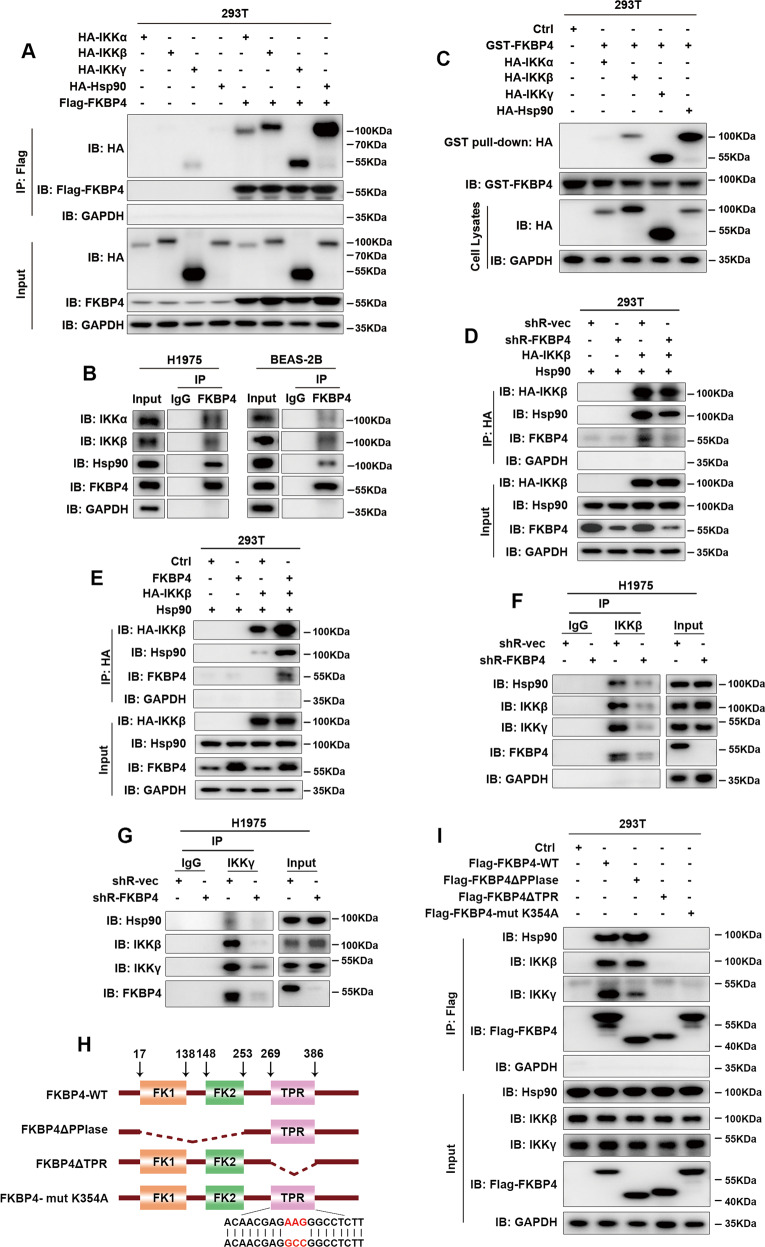
FKBP4 promotes Hsp90/IKK association and IKK complex assembly. **A** Co-IP analysis of 293T cells transfected with Flag-FKBP4 and the indicated plasmids, namely, HA-IKKα, HA-IKKβ, HA-IKKγ, and HA-Hsp90. FKBP4 interacted with all IKK complex subunits and Hsp90 under transfection conditions. **B** Co-IP analysis of endogenous FKBP4 interacting with endogenous IKKα, IKKβ, and Hsp90 in H1975 and BEAS-2B cells. **C** Pull down of HA-IKKβ, HA-IKKγ, and HA-Hsp90 by GST-tagged FKBP4. **D** Co-IP analysis of 293T cells with or without FKBP4 silencing and transfected with HA-IKKβ and Hsp90. FKBP4 knockdown impaired the interaction between Hsp90 and IKKβ under transfection conditions. **E** Co-IP analysis of 293T cells overexpressing or not overexpressing FKBP4 and transfected with HA-IKKβ and Hsp90. FKBP4 overexpression promoted the interaction between Hsp90 and IKKβ under transfection conditions. **F** Co-IP analysis showed that FKBP4 depletion blocked the interaction between Hsp90 and IKKβ in H1975 cells. **G** Co-IP analysis showed that FKBP4 depletion prevented the binding of IKKγ to IKKβ in H1975 cells. **H** Schematic illustration of FKBP4, showing the wild-type, truncation mutants (deleted PPIase or TPR domains), and point mutant (K354A) of FKBP4. **I** Co-IP analysis of the interaction between Hsp90/IKK and FKBP4 mutants in 293T cells transfected with the Flag-FKBP4 wild-type plasmid or plasmids harboring different mutations.

Since the role of Hsp90 in maintaining IKK kinase activity, we approached the study to investigate the interaction of FKBP4 with Hsp90/IKK proteins. As shown in Fig. 4A, FKBP4 bound to all three subunits of the IKK complex, IKKα, IKKβ, and IKKγ, as well as Hsp90 under the transfection conditions in 293T cells. Similar results were detected under endogenous conditions in H1975 and BEAS-2B cells (Fig. 4B). GST pull-down assays revealed that FKBP4 directly interacts with IKKβ, IKKγ, as well as Hsp90, while the interaction with IKKα was not detected. It is feasible that the interaction of FKBP4 with IKKα is IKKβ/IKKγ/Hsp90-mediated (Fig. 4C). Furthermore, we found that the association between IKK and Hsp90 was impaired in 293T cells transfected with shRNA FKBP4 (Fig. 4D), and vice versa (Fig. 4E). Consistent with these results, endogenous co-immunoprecipitation (co-IP) assays in H1975 cells further ascertained that FKBP4 knockdown blocked the interaction between IKK and Hsp90 (Fig. 4F). Moreover, FKBP4 knockdown dramatically reduced the binding of IKKγ to IKKβ, suggesting the role of this protein in facilitating IKK complex assembly, which was also required for phosphorylation of IKKβ (Fig. 4G)^28^.

Additionally, we constructed two truncated mutants of FKBP4 to define the interacting domains between FKBP4 and Hsp90/IKK (Fig. 4H). Our data revealed that the full-length FKBP4 and the PPIase domain-truncated mutants were capable of binding both Hsp90 and IKK, while the TPR domain-truncated mutant of FKBP4 failed to do so. Notably, the interaction between FKBP4 PPIase domain truncated form and IKKγ was significantly reduced compared with FKBP4 full length and IKKγ, suggesting the involvement of the PPIase domain in FKBP4/IKKγ interaction. To identify whether Hsp90 is required for the interaction between FKBP4 and IKK, we generated a TPR domain point mutant, Flag-FKBP4 K354A, which was unable to interact with Hsp90^30^. Interestingly, no interaction was detected between the mutant FKBP4 and IKK. It is feasible that the association between FKBP4 and IKK is dependent on Hsp90 existing (Fig. 4I).

### The association between FKBP4 and Hsp70/RelA impacts NF-κB activity

**Fig. 5:**
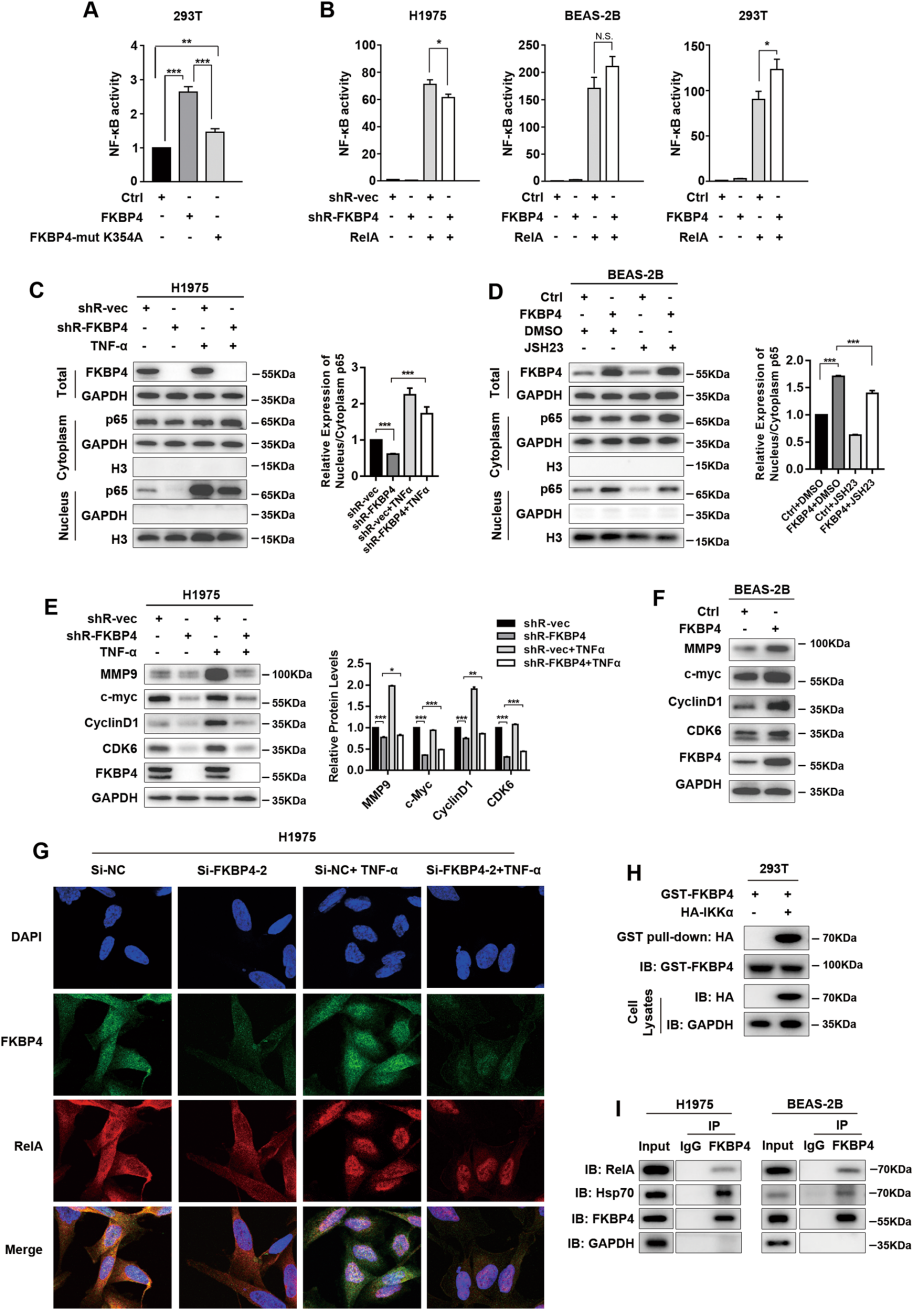
The interaction between FKBP4 and Hsp70/RelA impacts NF-κB activity. **A** Luciferase reporter assays of 293T cells transfected with wild-type FKBP4 or FKBP4 with a point mutant (K354A). **B** The effect of FKBP4 on RelA-induced NF-κB activity in H1975, BEAS-2B, and 293T cells. Luciferase reporter assays of these cells transfected with the indicated plasmids. **C**–**F** FKBP4 promotes the nuclear translocation of RelA and the expression of downstream targets of NF-κB. **C** Western blot analysis of H1975 cells transfected with FKBP4-targeting shRNA or control shRNA and treated with 10 ng/ml TNF-α for 24 h. **D** Western blot analysis of BEAS-2B cells transfected with FKBP4 vectors or control vectors and treated with 50 mM JSH23 for 24 h. **E** Western blot analysis of downstream targets of IKK/NF-κB signaling in H1975 cells with FKBP4 depletion and treated with 10 ng/ml TNF-α for 24 h. **F** Western blot analysis of downstream targets of IKK/NF-κB signaling in BEAS-2B cells overexpressing FKBP4. **G** Confocal microscope analysis of colocalization of FKBP4 and RelA upon TNF-α stimulation in H1975 cells. **H** Pull down of HA-RelA by GST-tagged FKBP4. **I** Co-IP analysis of endogenous FKBP4 interacting with endogenous RelA and Hsp70 in H1975 and BEAS-2B cells (*P < 0.01, **P < 0.01, ***P < 0.001).

Luciferase reporter assays were performed to dissect whether the FKBP4/Hsp90/IKK interaction is required for NF-κB activity. The results showed that the stimulatory action of 293T cells transfected with FKBP4 point mutant (K354A) was weakened compared with that of 293T cells transfected with wild-type FKBP4. In addition, the FKBP4 point mutant (K354A) still generated stimulatory action on NF-κB activity compared with the control cells (Fig. 5A). Based on these results, we wondered whether other molecular mechanisms were existing of FKBP4 to regulate NF-κB signaling. As shown in Fig. 5B, depletion of FKBP4 decreased RelA-induced NF-κB activity in H1975 cells, whereas overexpression of FKBP4 increased RelA-induced NF-κB activity in BEAS-2B and 293T cells. To analyze the effect of FKBP4 on NF-κB nuclear translocation, H1975 cells were transfected with FKBP4-targeting or control shRNA, followed by stimulation with TNF-α (Fig. 5C). In the control cells, the levels of RelA in nuclear cell extracts were increased in the presence of TNF-α. In FKBP4-depleted cells, the levels of RelA in the nucleus were significantly decreased compared with those in control cells. Additionally, upregulation of FKBP4 in BEAS-2B cells increased the level of RelA in the nucleus and restored the effect induced by JSH-23, which effectively blocked RelA nuclear translocation (Fig. 5D). Furthermore, the levels of downstream targets of NF-κB, including MMP9^31^, c-myc^32^, CyclinD1^33^, and CDK6^34^, were dramatically reduced after FKBP4 depletion in H1975 cells (Fig. 5E). In contrast, FKBP4 overexpression increased the levels of these downstream targets of NF-κB in BEAS-2B cells (Fig. 5F). As shown in Fig. 5G, FKBP4 knockdown did not reduce the levels of RelA in the nucleus, perhaps due to the low sensibility of immunofluorescence for quantitative analysis. But we found that the colocalization of FKBP4 with RelA was increased upon TNF-α stimulation, suggesting the potential existence of nuclear interactions^35^. Additionally, Hsp70 (HSPA1L), part of an endogenous complex with RelA^35,36^, was also identified to interact with FKBP4 using BioID technique (Supplementary Table S3). GST pull-down assays indicated the direct association between FKBP4 and Hsp70/RelA. Similar results were detected under endogenous conditions in H1975 and BEAS-2B cells. The binding of FKBP4 with Hsp70/RelA in LUAD was consistent with the previous study in 293T cells^35^.

Collectively, our data identified the regulatory mechanisms of RelA nuclear translocation by FKBP4 in LUAD as follows (Fig. 7J): (i) FKBP4 interacts with IKK subunits (IKKβ/γ), promotes Hsp90/IKKβ interaction, and IKK complex assembly, leading to IKKβ phosphorylation and IκBα proteasomal degradation. (ii) FKBP4 recruits dynein/dynactin motors and interacts with Hsp70/RelA, which favors RelA transport toward the nucleus on microtubule tracks^35^. Our collective data support that FKBP4/Hsp90/IKK and FKBP4/Hsp70/RelA complexes may promote NF-κB signaling and RelA nuclear translocation.

### High FKBP4 expression contributes to the proliferation, migration, and invasion of LUAD cells in vitro and in vivo

**Fig. 6:**
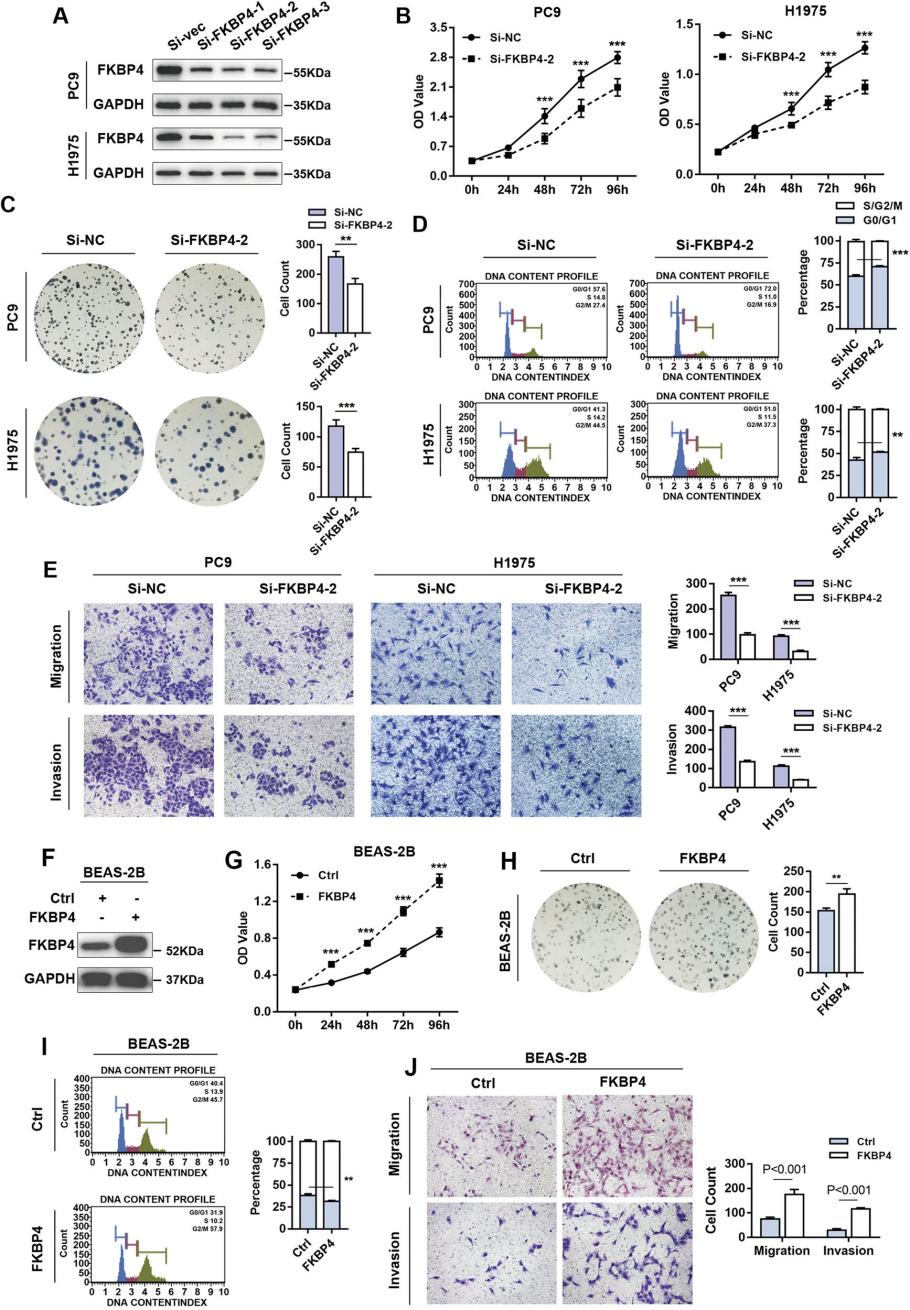
FKBP4 promotes the cell growth, metastasis, and invasion of LUAD cells and BEAS-2B cells. **A**–**E** PC9 and H1975 cells were transiently transfected with FKBP4 or negative control siRNAs. **A** Western blot analysis of successful FKBP4 knockdown in PC9 and H1975 cells. The effects of FKBP4 knockdown on cell proliferation were detected by CCK8 assay (**B**) and colony-formation assay (**C**). **D** Effects of FKBP4 knockdown on the cell cycle were detected by flow cytometry. **E** Effects of FKBP4 knockdown on metastasis and invasion were detected by Transwell assays ± Matrigel. **F**–**J** BEAS-2B cells were transiently transfected with FKBP4 or negative control plasmids. **F** Western blot analysis of successful FKBP4 overexpression in BEAS-2B cells. Effects of FKBP4 overexpression on cell proliferation were detected by CCK8 assay (**G**) and colony-formation assay (**H**). **I** Effects of FKBP4 overexpression on the cell cycle were detected by flow cytometry. **J** Effects of FKBP4 overexpression on metastasis and invasion were detected by Transwell assays ± Matrigel.

We performed a series of assays to identify the biological function of FKBP4 in LUAD. Due to the fact that FKBP4 was overexpressed in LUAD tissues, specific siRNAs were used to knock down the expression of FKBP4 in PC9 and H1975 cell lines (Fig. 6A). Interestingly, the proliferation (Fig. 6B) and colony-formation capabilities (Fig. 6C) were significantly decreased in FKBP4-inhibited PC9 and H1975 cells compared with their control counterparts. The percentage of FKBP4-depleted cells was significantly increased in G0/G1 phases coupled with reduction in G2/M phases (Fig. 6D). These results demonstrated that FKBP4 inhibition impaired cell proliferation by regulating the cell cycle in LUAD. Moreover, FKBP4 depletion might abrogate the migratory and invasive phenotypes of PC9 and H1975 cells (Fig. 6E).

In contrast, FKBP4 overexpression robustly increased the self-renewal and colony-formation abilities of BEAS-2B cells by promoting cell cycle progression (Fig. 6F–I). The migratory and invasive potentials were also elevated in BEAS-2B cells treated with the FKBP4 vector compared with those of cells treated with the corresponding control vector (Fig. 6J).

**Fig. 7:**
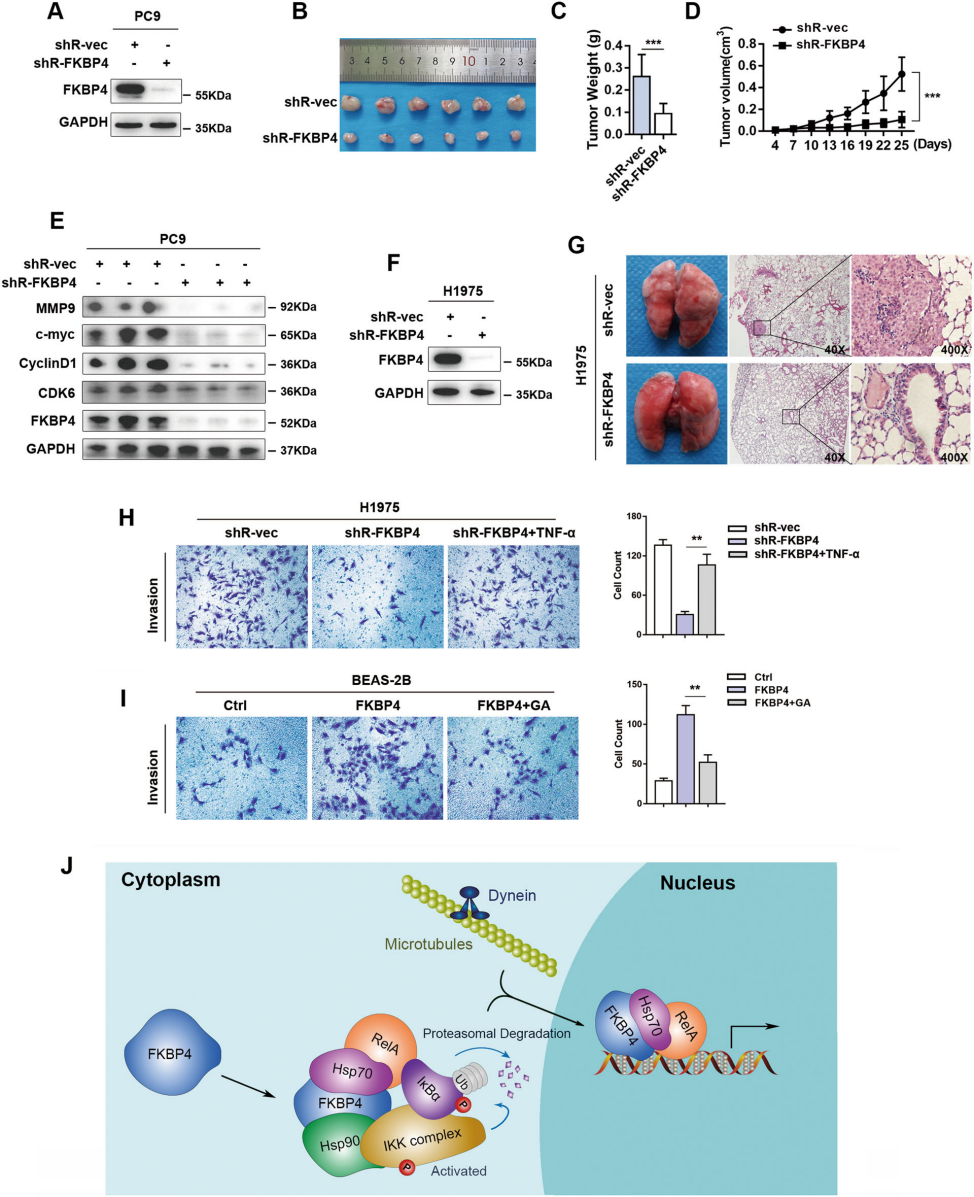
FKBP4 depletion suppresses cell proliferation and metastasis in vivo. **A**–**E** The xenograft tumor model was constructed by subcutaneous injection with PC9 cells expressing FKBP4 shRNA or the corresponding control cells. **A** Western blot analysis of PC9 cells successfully stably transfected with FKBP4 shRNA or control shRNA. **B** Images were photographed at the time of grafted removal (day 25). **C** Statistical analysis of the weights of grafted tumors on day 25. **D** Tumor volume was measured every 3 days before the mice were sacrificed. **E** Western blot analysis of targets downstream of IKK/NF-κB signaling in control and FKBP4-depleted tumors. **F**, **G** The tumor metastasis model was constructed by the tail vein injection of H1975 cells expressing FKBP4 shRNA or the corresponding control cells. **F** Western blot analysis of H1975 cells successfully stably transfected with FKBP4 shRNA or control shRNA. **G** Representative mouse lungs of each group were photographed at the time of mouse sacrifice, and H&E staining was performed to detect tumor metastasis. **H** Effects of FKBP4 knockdown with/without TNF-α on invasion were detected by Transwell assays + Matrigel. **I** Effects of FKBP4 knockdown with/without GA on invasion were detected by Transwell assays + Matrigel. **J** Schematic of the interactions of FKBP4 with both Hsp90/IKK and Hsp70/RelA.

To assess the effect of FKBP4 on tumor growth in vivo, PC9 cells stably transfected with FKBP4-targeting shRNA or control shRNA were subcutaneously injected into the flanks of nude mice (Fig. 7A). The volumes and weights of tumors excised from the FKBP4-knockdown group were strikingly reduced compared with those excised from the control group (Fig. 7B–D). Western blot assays verified the significant suppressive effect of FKBP4 inhibition on the regulation of downstream targets of NF-κB signaling (Fig. 7E). To gain insight into the involvement of FKBP4 in metastasis in vivo, H1975 cells stably transfected with shR-FKBP4 and shR-NC were injected into two groups of nude mice, respectively (Fig. 7F). Compared with mice injected with control cells, the mice injected with FKBP4-inhibited cells developed fewer lung metastatic nodules, as detected by histological H&E staining (Fig. 7G). Then, we investigated whether FKBP4 affects LUAD cell behaviors dependent on promoting NF-κB signaling. Results of the invasion assay showed that TNF-α stimulation could reverse the inhibition of FKBP4 depletion (Fig. 7H). Moreover, Hsp90 attenuator, geldanamycin (GA), decreased the invasive abilities of BEAS-2B cells induced by FKBP4 upregulation, suggesting that the association of Hsp90 with FKBP4 was important in FKBP4 biological functions (Fig. 7I).

Taken together, our results revealed that knocking down FKBP4 expression could attenuate the proliferation, metastasis, and invasion of LUAD cells in vitro and in vivo, perhaps explaining the finding that FKBP4 expression was correlated with a high pathological grade and TNM stages. In addition, the expression of FKBP4 might be important for lung epithelial cell tumorigenesis.

## Discussion

In the present study, TCGA database and IHC analysis were used to investigate the potential role of FKBP4 in LUAD. In two cohorts of LUAD patients, FKBP4 expression was elevated and positively correlated with the clinical TNM stage. Furthermore, high expression of FKBP4 was shown to decrease the OS of LUAD patients, indicating that FKBP4 may be a prognosis-associated marker in LUAD.

Many studies have demonstrated that FKBP4 and FKBP5 exhibited an antagonistic effect on the retrotransport mechanism of SRs (e.g., GR, MR, and PR^15,37–41^). In detail, FKBP5 is associated with the closed state of the Hsp90 dimer stabilized by the co-chaperone p23 and binds to empty SR in the cytoplasm^42^. Upon ligand binding, FKBP51 (unable to interact with dynein^37^) is exchanged by FKBP52, which recruits the dynein−dynactin motor complex to its PPIase domain and favors the receptor nuclear translocation on microtubule tracks^14,39^. A similar phenomenon has been described for the dynamic nucleocytoplasmic shuttling of NF-κB. In fibroblasts, FKBP4 interacts with RelA and is recruited to the promoter region of NF-κB target genes, while its association with Hsp90 is not required. On the contrary, FKBP5 counteracts the stimulatory effects of FKBP4 on NF-κB nuclear translocation^35^. Herein, we revealed the positive regulation of FKBP4 on IKK kinase activity, which was dependent on Hsp90 existing.

Hsp90 was identified as one of the FKBP4 proximal proteins by the BioID technique. As an alternative for conventional immunoprecipitation, the BioID approach could enrich for transient and/or low-affinity interactions within a 10-nm radius in the natural cellular environment^25,43,44^. The cells could be lysed under harsh buffer conditions since the biotin “tag” is covalently attached. In addition to SRs, Hsp90 has been shown to associate stoichiometrically with the IKK complex. Hsp90 contributes to the phosphorylation of IKKβ and/or shuttling of IKK to the plasma membrane^26^. We herein demonstrated that FKBP4 binds to Hsp90 and IKK subunits, and potentiated Hsp90−IKK interaction. Furthermore, FKBP4 supported a facilitation role in IKK complex assembly, which was also essential for IKKβ phosphorylation. By using two FKBP4-truncated mutants, lacking of PPIase or TPR domains, we determined that the TPR domain was required for the interaction with Hsp90/IKK, while the PPIase domain was not. Moreover, the PPIase domain of FKBP4 favors the interaction between this protein and IKKγ. We next introduced an FKBP4 point mutant in the TPR domain (K354A), which was unable to interact with Hsp90. The results indicated that the binding of FKBP4 with IKK is dependent on Hsp90. Additionally, our results supported that FKBP4 increased RelA-induced NF-κB activity and interacted with Hsp70/RelA. Thus, the association between FKBP4 and Hsp70/RelA was also important in the regulation of NF-κB signaling by FKBP4.

Due to the critical involvement of NF-κB signaling in multiple cancers, we assessed the effect of FKBP4 on malignant behavior of LUAD cells. Our data suggest that FKBP4 inhibition results in cell cycle arrest in G0/G1 phases and decreases the numbers of cells in G2/M phases. CyclinD1, which facilitates the G1/S transition, is a well-established target gene of NF-κB^33^. Furthermore, IKKβ has been shown to function as a mitotic regulator. It has been reported that IKKβ can impact Aurora A stability, CDK1 activity, and CyclinB1 degradation, thereby decreasing G2/M-phase arrest^45–47^. These results demonstrate that FKBP4 regulates cell proliferation by promoting cell cycle progression. In addition to cell growth, FKBP4 also contributes to metastasis as well as invasion in cellular and animal models of LUAD.

In summary, our study sheds some light on the critical role of FKBP4 in LUAD. FKBP4 integrates FKBP4/Hsp90/IKK with FKBP4/Hsp70/RelA complex, which subsequently potentiates the transcriptional activity and nuclear translocation of NF-κB, thereby enhancing proliferation and metastasis of LUAD. Activation of NF-κB is unambiguously linked to tumor development and therapeutic resistance. Therefore, inhibiting FKBP4 expression may be an efficient approach to treating LUADs exhibiting increased NF-κB activity.

## Materials and methods

### Tissue specimens

The tissue microarray (TMA, no. HLugA180Su05) was purchased from Shanghai Outdo Biotechnology Co., Ltd (Shanghai, China). It contained 94 specimens of LUAD tissues and 86 specimens of para-carcinoma tissues along with survival data (from 2005 to 2014).

### Immunohistochemistry and immunofluorescence

Formalin-fixed paraffin-embedded sections (4-μm thick) were mounted on silanized slides. After deparaffinization with xylene and rehydration with graded alcohol solutions, the slides were immersed in 10 mM citrate buffer (pH 8.0) and heat-treated for antigen retrieval. Endogenous peroxidase activity was quenched by the addition of 3% hydrogen peroxide. Then, the sections were incubated with an anti-FKBP4 antibody (1:400 dilution, Abcam, ab129097) overnight at 4 °C and then with a biotinylated secondary antibody. Standard DAB staining was performed to detect immunohistochemistry (IHC) targets. Scores were obtained by estimating the staining intensity (0 = none; 1 = mild; 2 = intermediate; 3 = intense) and the percentage of positive cells (0 = none; 1 = 1−25%; 2 = 26−50%; 3 = 51−75%; 4 = more than 75%). The intensity and percentage scores were multiplied to obtain the total score (the cut-point score = 6).

Cells were seeded into 24-well plates and fixed with 4% paraformaldehyde for 15 min. After permeabilization with 0.5% Triton X-100 and incubation with blocking buffer (normal goat serum), the cells were incubated with FKBP4 antibody and RelA antibody (CST, #6956) overnight at 4 °C. After 1 h of incubation with Alexa Fluor^®^ 555 (Abcam, ab 150086) and Alexa Fluor^®^ 647 (Abcam, ab150119), the cell nucleus was stained with DAPI and imaged by confocal microscopy (Olympus).

### Cell culture and transfection

The cell lines PC9, H1975, and BEAS-2B were purchased from the Cell Bank of the Chinese Academy of Sciences (Shanghai, China) and authenticated using STR profiling. The cells were cultured in PRMI-1640 medium (Gibco, MA, USA) supplemented with 10% fetal bovine serum (Gibco) and 1% penicillin−streptomycin (Solarbio, Beijing, China) at 37 °C in a humidified atmosphere with 5% CO_2_.

The small-interfering RNA (siRNA) targeting FKBP4 was synthesized by GenePharma (Shanghai, China). The following plasmids were purchased from BioSune (Shanghai, China): samples of plasmids encoding human Hsp90, IKKβ, and RelA; hemagglutinin (HA)-tagged IKKα, IKKβ, IKKγ, and Hsp90; Flag-tagged FKBP4 full-length (Flag-FKBP4-WT); Flag-tagged FKBP4-deleted PPIase domains (Flag-FKBP4ΔPPIase; 17-253 AA-truncated mutant); Flag-tagged FKBP4-deleted TPR domains (Flag-FKBP4ΔTPR; 269-386 AA-truncated mutant); Flag-tagged FKBP4 with a point mutant in the TPR domain (K354A). All transfections were performed with Lipofectamine 3000 (Invitrogen, MA, USA) according to the manufacturer’s instructions.

Lentiviruses carrying the FKBP4−RNA interference sequence (shR-FKBP4) or FKBP4-overexpression sequence were purchased from GeneChem (Shanghai, China). The stably transfected cells were further selected with puromycin (2 μg/ml) and validated by western blot.

### Luciferase reporter assay

To measure the transcriptional activity of NF-κB, cells were seeded in 24-well plates at a density of 10^4^ cells/well. After 24 h, the cells were further cotransfected with NF-κB-Luc, pRL-TK (Genomeditech, Shanghai, China), and the indicated plasmids. In 48 h, the cells were stimulated with TNF-α (10 ng/mL) for 6 h prior to being collected. Next, the ratio of Firefly to Renilla luciferase was detected by using the Dual-Luciferase Reporter Assay System (Promega, Shanghai, China) and defined as the relative luciferase activity.

### Western blot, co-IP, GST pull-down assays, and antibodies

Western blot assays were performed as described previously^48,49^, and the following antibodies were used for immunoblotting: anti-p-IKKα/β (Cell Signaling Technology (CST), #2697), anti-IKKβ (CST, #8943), anti-IKKα (CST, #11930), anti-p-IκBα (CST, #2859), anti-IκBα (CST, #4814), anti-RelA (CST, #8242), anti-p-RelA (CST, #3033), anti-GAPDH (Abcam, Cambridge, UK, ab181602), anti-FKBP4 (Abcam, ab129097), anti-CDK6 (CST, #13331), anti-MMP9 (CST, #13667), anti-CyclinD1 (CST, #2978), anti-c-Myc (CST, #13987), anti-Hsp90 (Abcam, ab203126), anti-Histone H3 (CST, #4499), and anti-HA-Tag (CST, #3724).

For co-IP, cells were incubated with lysis buffer (50 mM Tris-HCl, pH 7.5; 150 mM NaCl; 1% Triton X-100; 0.1% Na-deoxycholate; 1 mM ethylenediaminetetraacetic acid (EDTA); 1 mM dithiothreitol (DTT); 1:100 protease inhibitor cocktail). The following antibodies were used for co-IP: anti-FKBP4 (Abcam, ab230951), anti-IKKβ (Abcam, ab32135), anti-HA Tag (Sigma-Aldrich, MO, USA, 05-904), anti-FLAG M2 (Sigma-Aldrich, F1804), anti-rabbit IgG (CST, #2729), and anti-mouse IgG (Abcam, ab190475).

GST pull-down assays were performed with Pierce GST Protein Interaction Pull-Down Kit (Thermo Scientific, 21516) following the manufacturer’s suggestions. GST-pEGX-4T-1-FKBP4 was transformed into *coli*. BL21 (DE3), then using IPTG to induce protein expression. Bacterial lysates containing GST-FKBP4 were incubated with glutathione agarose at 4 °C for 1 h. After washing, GST-FKBP4 and the bound proteins were eluted from the beads using 10 mM glutathione elution buffer and subjected to gel analysis.

### BioID and mass spectrometry experiments

Eight 10-cm plates of H1975 cells stably transfected with HA-BirA* or HA-BirA*-FKBP4 were grown to 70% confluence prior to treatment with 50 μM biotin for 24 h. The cells were harvested and washed twice with cold PBS before cell lysis. Biotinylated proteins were isolated by affinity purification with streptavidin-labeled agarose beads as previously described^24,50^. Mass spectrometry assays were performed by Shanghai Applied Protein Technology (Shanghai, China). In addition, we used the MA-plot-based method with the Random Sampling model (MARS) to identify the high-confidence proximal interactors of FKBP4.

### Cell proliferation and colony-formation assays

Cells were seeded into 96-well plates at 4 × 10^3^ cells/well, and the proliferation of these cells was detected using Cell Counting Kit-8 (CCK-8; Dojindo, Kumamoto, Japan) according to the manufacturer’s protocol.

For the colony-formation assay, cells were plated in six-well plates at 1000 cells/well. In the second week, colonies were stained with 0.1% crystal violet.

### Cell cycle and migration/invasion assays

Treated cells were stained with Muse Cell Cycle Reagent (Merck, Darmstadt, Germany) according to the manufacturer’s instructions and then further detected by a Muse Cell Analyzer (Merck) to assess the cell cycle distribution. Migration and invasion assays were performed as previously described^49^.

### In vivo tumor growth and metastasis assays

The animal studies were approved by the Medical Ethics Committee of Shandong Provincial Hospital. Male nude mice (5 weeks old) were purchased from Beijing HFK Biotechnology Co., Ltd. (Beijing, China).

To induce ectopic tumors, a number of 10^7^ PC9 stably transfected cells suspended in 100 µl of medium were injected subcutaneously (*n* = 10 per group). The primary tumor size was measured from the outside of the mouse’s skin every 3 days and calculated by the following formula: volume = 1/2 × length × width^2^. After 25 days, tumor nodules were surgically excised for further analysis. For the metastasis assay, a total of 5 × 10^5^ H1975 stably transfected cells suspended in 100 μl of medium were injected into the tail vein (*n* = 5 per group). Mice were killed after 8 weeks, and their lungs were harvested for the evaluation of tumor metastasis by hematoxylin and eosin (H&E) staining.

### Transcriptome sequencing

Total RNA was extracted from H1975 cells using TRIzol reagent (TaKaRa, Kyoto, Japan). The samples were run on 1% agarose gels and evaluated using the NanoPhotometer spectrophotometer (IMPLEN, CA, USA). The sequencing library was prepared by NEBNext UltraTM RNA Library Prep Kit for Illumina (NEB, USA) according to the standard protocols. The RNA-seq experiments were performed by Novogene (Tianjin, China). The DEGseq2 R package (1.16.1) was applied to determine differential expression between the two groups based on fragments per kilobase per million read (FPKM) values. The heat map was generated using Novomagic.

### Statistical analysis

SPSS version 22.0 (IL, USA) and GraphPad Prism 7.00 (CA, USA) were used for statistical analysis. The chi-square test was performed to analyze the association between FKBP4 expression and clinicopathologic variables. Kaplan−Meier analysis and the corresponding log-rank tests were used to estimate survival curves. Uni- and multivariate Cox regression models were utilized to evaluate the potential prognostic biomarkers. ROC analysis was employed to compare the sensitivities and specificities of the survival predictions according to different risk factors. The cut-off values of FKBP4 expression and TNM stages were calculated by MedCalc 15.2.2 software (Mariakerke, Belgium). Student’s *t*-test and one-way ANOVA were applied to evaluate statistical variance between groups.

## Supplementary information

Supplementary Material

## Data Availability

GEO accession ID for the RNA-seq data is GSE157826.
