# Supplementary material for: FKBP4 integrates FKBP4/Hsp90/IKK with FKBP4/Hsp70/RelA complex to promote lung adenocarcinoma progression via IKK/NF-κB signaling
Source: Cell Death Dis. 2021 Jun 10;12(6):602. doi: 10.1038/s41419-021-03857-8 (PMC8192522; doi:10.1038/s41419-021-03857-8)
Supplement: Supplementary file 1 — Supplementary Material [file 41419_2021_3857_MOESM1_ESM.pdf]

Supplementary Material

Supplementary Figure and Table Legends

Supplementary Figure S1. (A) The mRNA levels of FKBP4 in human LUAD were remarkably elevated compared with those in normal tissues, as determined from three independent datasets in the Oncomine database. (B) ROC analysis of OS using the TNM stage and FKBP4 expression.

Table S1. Overall survival data and FKBP4 FPKM values of LUAD patients based on TCGA database

Table S2. RNA-seq data of IKK/NF-κB pathway related genes in the control and FKBP4-knockdown groups

Table S3. FKBP4 BioID-based interactome.

Figure S1

A

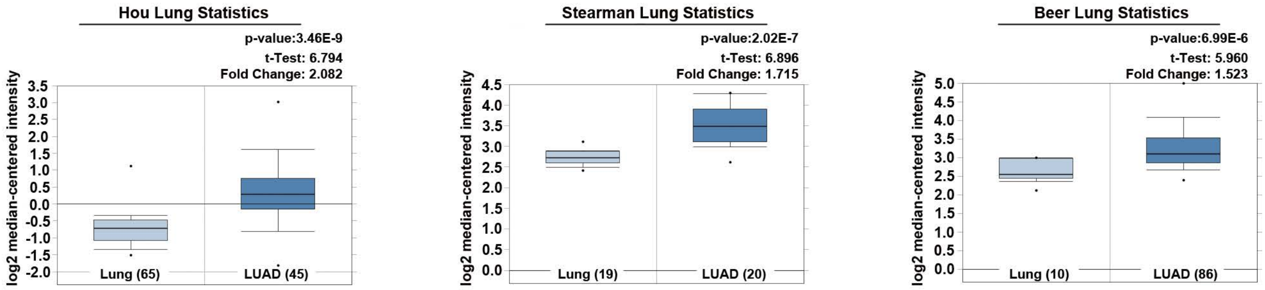

B

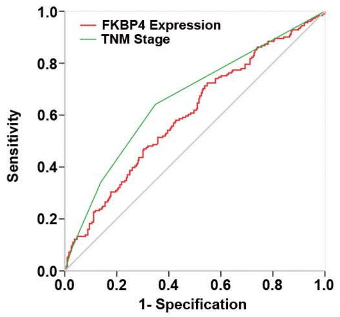

	FKBP4 Expression	TNM Stage
Cut-off value	10.8319	Ⅰ stage
Area Under the Curve (AUC)	0.602	0.663
Sensitivity	72.68	64.09
Sepecification	45.03	65.19

Table S1

High Expression of FKBP4			
sampleID	FKBP4 expression	_OS（days）	vital_status
TCGA-55-6985-01	10.9049	1233	LIVING
TCGA-55-6975-01	11.7486	118	DECEASED
TCGA-55-6987-01	12.1147	2137	LIVING
TCGA-55-8204-01	11.2982	515	LIVING
TCGA-55-8302-01	12.2892	478	LIVING
TCGA-55-7570-01	12.0361	824	LIVING
TCGA-55-8299-01	10.9505	469	DECEASED
TCGA-55-7907-01	11.3473	343	DECEASED
TCGA-55-8301-01	12.4307	534	LIVING
TCGA-55-8203-01	11.4864	547	LIVING
TCGA-49-4490-01	11.4736	385	DECEASED
TCGA-55-7576-01	11.9737	670	LIVING
TCGA-55-8616-01	10.9786	48	LIVING
TCGA-55-7903-01	12.217	567	LIVING
TCGA-55-7725-01	10.9459	442	LIVING
TCGA-55-6986-01	10.8378	3261	LIVING
TCGA-55-1595-01	11.6506	1479	LIVING
TCGA-95-7043-01	11.9813	503	DECEASED
TCGA-73-4668-01	11.0541	467	LIVING
TCGA-91-6831-01	11.9569	310	LIVING
TCGA-50-5944-01	11.0566	1750	LIVING
TCGA-62-8397-01	11.9766	1289	LIVING
TCGA-55-6984-01	12.435	760	DECEASED
TCGA-55-6972-01	10.9391	1632	DECEASED
TCGA-55-7726-01	12.2385	652	LIVING
TCGA-99-8033-01	13.4089	656	DECEASED
TCGA-69-7764-01	11.2313	414	LIVING
TCGA-69-8453-01	11.2519	813	LIVING
TCGA-55-8094-01	12.7685	541	LIVING
TCGA-73-4666-01	11.9013	800	LIVING
TCGA-78-7220-01	11.7982	807	DECEASED
TCGA-55-6980-01	11.7139	2109	LIVING
TCGA-55-7913-01	11.0292	561	DECEASED
TCGA-55-8085-01	12.2145	904	LIVING
TCGA-62-8394-01	10.9975	139	DECEASED
TCGA-38-4625-01	12.2078	2973	LIVING
TCGA-38-4631-01	12.8958	354	DECEASED
TCGA-78-7162-01	11.2575	3169	DECEASED
TCGA-55-8614-01	10.9494	536	LIVING
TCGA-55-7724-01	11.9314	705	LIVING
TCGA-78-7167-01	11.0384	2681	DECEASED
TCGA-55-6978-01	11.4892	176	DECEASED
TCGA-75-6205-01	11.177		DECEASED
TCGA-75-5147-01	11.7294	1333	LIVING
TCGA-05-4420-01	12.2648	912	LIVING
TCGA-38-4632-01	12.0683	1357	DECEASED
TCGA-55-6981-01	11.8187	1379	DECEASED
TCGA-78-8640-01	12.0452	7062	LIVING
TCGA-78-7160-01	11.8917	697	DECEASED
TCGA-38-4628-01	12.4946	1492	DECEASED
TCGA-49-4506-01	12.759	999	DECEASED
TCGA-J2-8194-01	11.092	724	LIVING
TCGA-55-7911-01	11.7376	537	LIVING
TCGA-95-7562-01	11.0914	87	DECEASED
TCGA-78-7146-01	13.1614	173	DECEASED
TCGA-97-8179-01	11.8865	435	LIVING
TCGA-55-7995-01	11.9081	889	LIVING
TCGA-64-5815-01	11.1083	866	LIVING
TCGA-78-8655-01	11.0021	2360	LIVING
TCGA-75-6211-01	11.5414		DECEASED
TCGA-35-4123-01	11.2973	182	LIVING
TCGA-91-6836-01	11.5425	417	LIVING
TCGA-55-1596-01	11.2657	2065	LIVING
TCGA-50-5936-01	12.0691	257	DECEASED
TCGA-05-4430-01	11.1376	761	LIVING
TCGA-78-7150-01	12.0938	666	DECEASED
TCGA-50-6591-01	11.6158	119	DECEASED
TCGA-78-7152-01	11.2621	1215	DECEASED
TCGA-55-8096-01	10.9355	719	DECEASED
TCGA-55-8615-01	11.7105	446	LIVING
TCGA-78-7155-01	11.1791	1171	DECEASED
TCGA-05-4382-01	11.5316	607	LIVING
TCGA-67-4679-01	11.0047	448	LIVING
TCGA-05-5715-01	11.2356	62	LIVING
TCGA-69-7979-01	11.1755	408	LIVING
TCGA-55-6712-01	11.2557	171	DECEASED
TCGA-78-7148-01	11.8782	626	DECEASED
TCGA-49-4487-01	11.5337	855	DECEASED
TCGA-78-7154-01	12.5131	593	DECEASED
TCGA-05-4405-01	10.9471	610	LIVING
TCGA-73-4677-01	11.2636	38	DECEASED
TCGA-91-6829-01	11.5033	1258	DECEASED
TCGA-78-7166-01	11.7894	258	DECEASED
TCGA-75-7031-01	10.9626		LIVING
TCGA-44-6779-01	11.3188	500	DECEASED
TCGA-44-5644-01	11.958	863	LIVING
TCGA-64-5781-01	11.0375	1559	LIVING

TCGA-50-5941-01	11.2745	1474	LIVING
TCGA-62-8398-01	12.4067	444	DECEASED
TCGA-69-7765-01	11.2095	165	LIVING
TCGA-78-7542-01	12.455	321	DECEASED
TCGA-05-5428-01	11.76	670	LIVING
TCGA-64-1679-01	11.3166	2488	LIVING
TCGA-55-5899-01	11.6162	930	LIVING
TCGA-69-7978-01	10.8742	134	LIVING
TCGA-55-6543-01	11.0209	435	LIVING
TCGA-05-5429-01	11.2016	275	DECEASED
TCGA-55-8620-01	11.3157	375	DECEASED
TCGA-55-8508-01	11.5168	617	LIVING
TCGA-44-7662-01	10.9775	218	LIVING
TCGA-95-7567-01	12.1965	568	LIVING
TCGA-62-8399-01	11.01	2696	LIVING
TCGA-05-4410-01	11.1151		LIVING
TCGA-44-7671-01	11.01	889	LIVING
TCGA-44-2662-01	11.3745	1280	LIVING
TCGA-05-5420-01	11.4898	457	LIVING
TCGA-35-4122-01	12.3642	225	LIVING
TCGA-64-1678-01	11.904	1189	LIVING
TCGA-78-7149-01	11.2523	3940	LIVING
TCGA-69-8255-01	13.2367	129	LIVING
TCGA-44-7660-01	11.8919	592	LIVING
TCGA-86-6851-01	11.7933	179	LIVING
TCGA-49-4507-01	12.1761	268	DECEASED
TCGA-05-4395-01	13.6588	0	DECEASED
TCGA-64-1677-01	11.1346	628	DECEASED
TCGA-55-8513-01	11.4156	791	LIVING
TCGA-62-8395-01	11.2452	1216	LIVING
TCGA-55-7994-01	11.0886	603	LIVING
TCGA-67-3773-01	10.8347	427	LIVING
TCGA-75-6207-01	12.3539		DECEASED
TCGA-05-4415-01	13.1093	91	DECEASED
TCGA-91-6848-01	11.8419	224	LIVING
TCGA-78-7158-01	12.0426	179	DECEASED
TCGA-78-7143-01	10.8344	4961	DECEASED
TCGA-55-8506-01	11.1766	11	LIVING
TCGA-78-7156-01	11.9619	976	DECEASED
TCGA-97-7937-01	11.7083	564	LIVING
TCGA-55-6970-01	11.6656	464	DECEASED
TCGA-86-7954-01	11.0307	605	LIVING
TCGA-78-7536-01	11.8235	244	DECEASED
TCGA-78-8660-01	11.982	321	DECEASED
TCGA-05-4244-01	11.6474		LIVING
TCGA-05-4424-01	12.292	913	LIVING
TCGA-69-7760-01	11.2289	202	LIVING
TCGA-55-8511-01	12.2696	552	LIVING
TCGA-55-8091-01	11.587	600	LIVING
TCGA-55-8205-01	11.1256	599	LIVING
TCGA-49-6745-01	10.8557	522	LIVING
TCGA-05-4433-01	11.2864	730	LIVING
TCGA-50-6673-01	11.0091	22	DECEASED
TCGA-50-5051-01	11.5927	478	DECEASED
TCGA-50-5930-01	10.8767	282	DECEASED
TCGA-44-2668-01	11.2546	761	DECEASED
TCGA-44-5643-01	14.4638	1013	LIVING
TCGA-78-8648-01	11.117	1209	DECEASED
TCGA-55-8505-01	11.5829	440	LIVING
TCGA-73-4659-01	11.4455	711	DECEASED
TCGA-49-4501-01	10.9575	1421	DECEASED
TCGA-95-7947-01	11.9257	477	LIVING
TCGA-05-5425-01	11.2964	882	LIVING
TCGA-05-4418-01	12.9995	274	DECEASED
TCGA-73-4670-01	13.5681	131	LIVING
TCGA-44-7659-01	11.3737	691	LIVING
TCGA-99-8025-01	11.453	1060	LIVING
TCGA-99-7458-01	11.0001	747	LIVING
TCGA-50-5072-01	13.0337	250	DECEASED
TCGA-91-8499-01	12.0275	36	LIVING
TCGA-75-5126-01	11.9156		LIVING
TCGA-95-7948-01	12.2815	476	LIVING
TCGA-35-5375-01	11.4952	264	LIVING
TCGA-99-8032-01	11.7884	44	LIVING
TCGA-44-2659-01	11.3121	1367	LIVING
TCGA-78-7540-01	11.5065	1197	DECEASED
TCGA-05-4434-01	12.026	457	DECEASED
TCGA-38-4629-01	10.9105	864	DECEASED
TCGA-05-4389-01	11.6116	1369	LIVING
TCGA-95-7944-01	12.5785	377	LIVING
TCGA-64-5775-01	12.7283	62	DECEASED
TCGA-69-7761-01	11.4449	186	LIVING
TCGA-05-4417-01	11.8402	455	LIVING
TCGA-78-7145-01	11.1238	826	DECEASED
TCGA-05-5423-01	10.9628	151	LIVING
TCGA-95-8494-01	10.8567	84	LIVING
TCGA-44-7661-01	11.2937	557	DECEASED
TCGA-50-5044-01	11.8268	624	DECEASED
TCGA-55-8208-01	11.0788	674	LIVING
TCGA-50-5932-01	10.9362	1235	DECEASED
TCGA-49-4514-01	11.8037	1700	LIVING
TCGA-73-4658-01	11.3071	1600	DECEASED

TCGA-80-5607-01	11.0607		LIVING
TCGA-75-7027-01	12.3241	3059	LIVING
TCGA-73-4676-01	10.8501	281	DECEASED
TCGA-49-6767-01	11.5816	677	LIVING
TCGA-44-7670-01	11.0855	882	LIVING
TCGA-05-4398-01	12.8491	1431	LIVING
TCGA-64-1676-01	11.0326	1728	LIVING
TCGA-44-6776-01	11.2458	2616	LIVING
TCGA-49-4505-01	11.5273	428	DECEASED
TCGA-44-6145-01	11.0862	595	LIVING
TCGA-50-5946-01	11.3183	1617	LIVING
TCGA-97-8174-01	10.8814	164	DECEASED
TCGA-05-4422-01	11.6338	365	LIVING
TCGA-50-5066-01	11.5998	1442	LIVING
TCGA-50-5066-02	12.2044	1442	LIVING
TCGA-91-6849-01	10.878	35	LIVING
TCGA-05-4403-01	11.8677	578	LIVING
TCGA-50-6594-01	11.0618	370	DECEASED
TCGA-05-4402-01	12.0163	244	DECEASED
TCGA-73-4675-01	11.3245	922	DECEASED
TCGA-91-6830-01	11.1869	60	LIVING
TCGA-69-7980-01	10.9981	362	LIVING
TCGA-49-4486-01	11.2314	2318	DECEASED
TCGA-50-6590-01	12.565	1288	DECEASED
TCGA-86-8281-01	11.0447		LIVING
TCGA-38-4630-01	12.1321	1073	DECEASED
TCGA-05-4396-01	10.8687	303	DECEASED
TCGA-93-8067-01	12.3503	186	LIVING
TCGA-75-5125-01	11.7961	2027	DECEASED
TCGA-86-8076-01	11.2953	993	LIVING
TCGA-86-8358-01	11.3219	653	LIVING
TCGA-44-7667-01	11.3153	1097	LIVING
TCGA-86-8054-01	12.4626	1148	LIVING
TCGA-05-4427-01	11.1261	791	LIVING
TCGA-50-5933-01	11.6229	2393	DECEASED
TCGA-50-6595-01	11.4434	189	DECEASED
TCGA-97-8172-01	11.0465	545	LIVING
TCGA-50-5931-01	12.9407	434	DECEASED
TCGA-50-5045-01	11.1197	2174	DECEASED
TCGA-86-8073-01	10.939	740	LIVING
TCGA-05-4390-01	11.4768	1126	LIVING
TCGA-44-7669-01	13.4378	574	DECEASED
TCGA-55-1592-01	11.5427	701	DECEASED
TCGA-86-8075-01	11.2657	694	DECEASED
TCGA-86-7701-01	12.0542	947	LIVING
TCGA-86-7711-01	12.3856	1046	DECEASED
TCGA-44-6777-01	11.4283	987	DECEASED
TCGA-86-6562-01	11.097	376	DECEASED
TCGA-50-5946-02	11.4729	1617	LIVING
TCGA-97-8171-01	12.412	568	LIVING
TCGA-75-6214-01	12.3083	1115	DECEASED
TCGA-44-2666-01	10.8329	97	DECEASED
TCGA-71-6725-01	11.2897	256	LIVING
TCGA-44-4112-01	12.4661	808	DECEASED
TCGA-50-6592-01	12.2074	777	DECEASED
TCGA-49-4488-01	11.4085	869	DECEASED
TCGA-50-6597-01	11.1649	1268	DECEASED
TCGA-86-7955-01	12.2495	1072	LIVING
TCGA-97-8176-01	12.1561	468	DECEASED
TCGA-49-6744-01	10.8666	1683	LIVING
TCGA-49-6742-01	11.4893	488	DECEASED
TCGA-86-8585-01	11.4648	353	LIVING
TCGA-86-8672-01	11.4551	19	DECEASED
TCGA-86-8669-01	11.6628	938	LIVING
TCGA-49-4512-01	11.5949	905	DECEASED
TCGA-86-8074-01	11.0406	24	LIVING
TCGA-49-6761-01	11.9505	354	LIVING
TCGA-86-8674-01	11.3759	806	LIVING
TCGA-86-8359-01	10.9346	444	DECEASED
TCGA-49-4494-01	11.697	1081	DECEASED
TCGA-75-6212-01	11.3523	1516	DECEASED
TCGA-44-8119-01	10.8792	285	LIVING
TCGA-86-8673-01	11.8137	862	LIVING
TCGA-91-7771-01	11.2383	492	LIVING
TCGA-86-7713-01	11.4989	1157	LIVING
TCGA-86-8055-01	11.1726	124	DECEASED
TCGA-49-AARO-01	10.9222	3759	LIVING
TCGA-L9-A5IP-01	11.6507	58	DECEASED
TCGA-53-7624-01	12.8053	1043	DECEASED
TCGA-73-A9RS-01	12.627	340	DECEASED
TCGA-L4-A4E5-01	11.6786	578	LIVING
TCGA-86-A4D0-01	12.005	116	DECEASED
TCGA-NJ-A4YF-01	12.0969	2161	LIVING
TCGA-4B-A93V-01	10.8592	300	DECEASED
TCGA-NJ-A4YP-01	11.4878	50	LIVING
TCGA-78-8662-01	11.505	3361	DECEASED
TCGA-62-A46R-01	10.8756	1725	DECEASED
TCGA-55-A493-01	12.6154	28	LIVING
TCGA-MP-A4TK-01	10.9093	582	DECEASED
TCGA-49-AARN-01	10.9206	1135	DECEASED
TCGA-NJ-A55O-01	11.3767	13	LIVING
TCGA-86-A4JF-01	12.06	737	DECEASED

TCGA-MP-A4TE-01	12.152	896	DECEASED
TCGA-MN-A4N4-01	11.0366	1175	LIVING
TCGA-49-AAR0-01	11.2813	4765	LIVING
TCGA-35-3615-01	11.5387	14	LIVING
TCGA-MP-A4TF-01	11.9981	336	DECEASED
TCGA-L9-A444-01	10.9709	307	LIVING
TCGA-69-A59K-01	11.2686	522	LIVING
TCGA-44-3918-01	11.304	1036	LIVING
TCGA-MN-A4N1-01	11.6696	827	LIVING
TCGA-97-A4M0-01	12.0542	652	LIVING
TCGA-J2-A4AD-01	11.3252	550	DECEASED
TCGA-MP-A4SY-01	11.5263	1501	DECEASED
TCGA-49-AAR9-01	11.7814	260	DECEASED
TCGA-55-A494-01	12.5351	481	LIVING
TCGA-95-A4VN-01	11.524	553	LIVING
TCGA-L9-A443-01	11.7397	193	DECEASED
TCGA-44-6146-01	11.1825	728	LIVING
TCGA-L9-A8F4-01	11.5251	476	LIVING
TCGA-62-A471-01	12.1773	1246	LIVING
TCGA-NJ-A4YG-01	11.3173	2261	LIVING
TCGA-62-A46O-01	11.3354	1454	DECEASED
TCGA-95-A4VP-01	10.9311	605	LIVING
TCGA-J2-A4AG-01	11.1696	988	LIVING
TCGA-44-A4SU-01	10.9362	409	DECEASED
TCGA-NJ-A55R-01	11.7263	603	LIVING
TCGA-MP-A4SV-01	11.9115	2620	DECEASED
TCGA-MP-A4T4-01	11.1811	2617	DECEASED
TCGA-MP-A4T8-01	11.9794	161	DECEASED
TCGA-NJ-A4YQ-01	11.1037	1432	LIVING
TCGA-55-A48Y-01	11.7965	630	LIVING
TCGA-93-A4JO-01	10.8503	33	DECEASED
TCGA-55-A492-01	11.5896	596	LIVING
TCGA-62-A472-01	12.4874	910	LIVING
TCGA-MP-A4TD-01	10.8668	307	DECEASED
TCGA-62-A46U-01	11.1979	2067	LIVING
TCGA-MP-A4TI-01	11.5354	429	DECEASED
TCGA-44-A4SS-01	11.8137	415	LIVING
TCGA-L9-A50W-01	10.9014	442	DECEASED
TCGA-MP-A4T7-01	11.748	167	DECEASED
TCGA-MP-A4TA-01	12.5008	950	DECEASED
TCGA-91-A4BD-01	11.1776	603	LIVING
TCGA-44-A47A-01	11.2156	466	LIVING
TCGA-86-A456-01	11.4031	896	LIVING
TCGA-55-A490-01	12.2943	99	DECEASED
TCGA-38-A44F-01	11.3063	133	LIVING
TCGA-55-A491-01	10.8431	626	LIVING
TCGA-NJ-A4YI-01	11.4066	4	DECEASED
TCGA-55-A4DF-01	11.1885	614	DECEASED

Low Expression of FKBP4

sampleID	FKBP4 expression	_OS（days）	vital_status
TCGA-55-7283-01	10.4267	609	LIVING
TCGA-55-8089-01	10.8046	702	DECEASED
TCGA-55-7816-01	10.5104	468	DECEASED
TCGA-55-7281-01	10.7071	872	LIVING
TCGA-55-7914-01	10.4826	187	DECEASED
TCGA-55-8097-01	10.3047	476	LIVING
TCGA-44-2655-01	10.59	1324	LIVING
TCGA-55-8514-01	10.4442	520	LIVING
TCGA-67-3774-01	9.8111	385	LIVING
TCGA-55-7227-01	10.3214	952	DECEASED
TCGA-55-6982-01	10.5347	995	DECEASED
TCGA-55-6983-01	10.5967	2823	LIVING
TCGA-67-6215-01	10.3513	174	LIVING
TCGA-55-7727-01	10.653	119	LIVING
TCGA-44-3919-01	10.4603	1026	DECEASED
TCGA-75-7030-01	10.1276		LIVING
TCGA-75-7025-01	10.3907	3305	LIVING
TCGA-91-6840-01	10.6176	372	LIVING
TCGA-55-6968-01	10.7639	1293	DECEASED
TCGA-97-7547-01	10.5419	1965	LIVING
TCGA-62-8402-01	10.8001	1498	DECEASED
TCGA-55-8621-01	10.5898	515	LIVING
TCGA-55-8092-01	10.7161	154	DECEASED
TCGA-55-7815-01	10.1816	773	LIVING
TCGA-75-6206-01	10.818	2590	LIVING
TCGA-53-7813-01	10.5192	424	LIVING
TCGA-95-7039-01	10.599	1272	LIVING
TCGA-69-7974-01	10.6056	184	LIVING
TCGA-44-2656-01	10.7041	1429	LIVING
TCGA-64-1681-01	10.6966	1167	DECEASED
TCGA-73-4662-01	10.6626	2515	LIVING
TCGA-05-4384-01	10.6292	426	LIVING
TCGA-75-5122-01	10.0424		DECEASED
TCGA-75-5146-01	10.6753	2368	LIVING
TCGA-69-7973-01	9.9935	230	LIVING
TCGA-55-7910-01	10.8227	1040	LIVING
TCGA-49-4510-01	9.5563	896	DECEASED
TCGA-55-6969-01	10.6512	1239	LIVING
TCGA-55-8206-01	9.7651	888	LIVING
TCGA-55-8087-01	9.5642	462	LIVING

TCGA-71-8520-01	10.1688	210	DECEASED
TCGA-55-7574-01	10.3667	995	DECEASED
TCGA-05-4397-01	10.7099	731	DECEASED
TCGA-78-7147-01	10.5378	586	DECEASED
TCGA-69-7763-01	10.5209	690	LIVING
TCGA-67-3770-01	10.6604	610	LIVING
TCGA-78-7537-01	9.7448	1622	DECEASED
TCGA-55-7573-01	10.1183	487	LIVING
TCGA-86-8671-01	10.2473	839	LIVING
TCGA-95-8039-01	10.4315	830	LIVING
TCGA-55-8207-01	10.2719	977	LIVING
TCGA-55-7284-01	10.8042	243	DECEASED
TCGA-78-7539-01	10.7659	791	LIVING
TCGA-67-3772-01	10.3319	573	LIVING
TCGA-55-8512-01	10.1391	607	DECEASED
TCGA-55-8507-01	10.408	418	LIVING
TCGA-38-4626-01	10.3518	3674	LIVING
TCGA-44-8120-01	10.1809	260	LIVING
TCGA-73-7498-01	10.596	1189	LIVING
TCGA-55-6979-01	10.1375	237	DECEASED
TCGA-44-6778-01	10.725	1864	LIVING
TCGA-55-6642-01	9.8736	2449	LIVING
TCGA-55-7728-01	9.7643	704	LIVING
TCGA-78-7153-01	9.9291	3635	LIVING
TCGA-J2-8192-01	10.7971	739	LIVING
TCGA-05-4432-01	9.8741	761	LIVING
TCGA-50-5942-01	9.5525	1847	LIVING
TCGA-44-6147-01	10.2502	845	LIVING
TCGA-78-7633-01	10.5791	1528	DECEASED
TCGA-78-7161-01	10.5837	291	DECEASED
TCGA-44-2657-01	10.1708	1351	LIVING
TCGA-93-7347-01	10.8257	683	LIVING
TCGA-55-8090-01	9.8921	598	DECEASED
TCGA-75-6203-01	10.5727		LIVING
TCGA-44-8117-01	10.2288	385	LIVING
TCGA-55-6971-01	10.3412	1400	LIVING
TCGA-78-7159-01	9.7472	1974	LIVING
TCGA-91-6847-01	10.8106	842	LIVING
TCGA-05-4425-01	10.177	669	LIVING
TCGA-38-7271-01	10.6711	800	DECEASED
TCGA-67-6217-01	10.4567	422	LIVING
TCGA-91-6835-01	10.1634	79	LIVING
TCGA-78-7535-01	10.4525	949	DECEASED
TCGA-55-8510-01	10.2779	539	LIVING
TCGA-67-6216-01	10.0926	141	LIVING
TCGA-69-8253-01	10.7278	426	LIVING
TCGA-64-5778-01	10.4945	1305	LIVING
TCGA-78-7163-01	10.7862	7248	LIVING
TCGA-50-8457-01	10.7032	1125	LIVING
TCGA-64-1680-01	10.7158	1126	LIVING
TCGA-50-8459-01	9.8415	1119	LIVING
TCGA-55-8619-01	10.3376	416	LIVING
TCGA-97-7938-01	9.7866	18	DECEASED
TCGA-97-7546-01	10.1956	1285	LIVING
TCGA-05-4250-01	10.4736	121	DECEASED
TCGA-49-6743-01	10.7581	1621	LIVING
TCGA-50-5939-01	10.5247	460	DECEASED
TCGA-80-5611-01	9.8837	2595	LIVING
TCGA-80-5608-01	10.4633	2832	LIVING
TCGA-99-8028-01	10.7136	1118	LIVING
TCGA-50-7109-01	10.223	308	DECEASED
TCGA-44-5645-01	9.693	852	LIVING
TCGA-64-5779-01	10.8076	864	LIVING
TCGA-97-7941-01	10.8319	484	LIVING
TCGA-44-3396-01	10.2453	1130	LIVING
TCGA-50-8460-01	10.8085	829	LIVING
TCGA-50-6593-01	10.4245	336	DECEASED
TCGA-50-5068-01	9.9935	1499	DECEASED
TCGA-64-5774-01	10.5636	2676	LIVING
TCGA-86-8056-01	9.0954	139	LIVING
TCGA-38-4627-01	10.4958	1147	DECEASED
TCGA-05-4249-01	9.9528	1523	LIVING
TCGA-69-8254-01	9.3813	409	LIVING
TCGA-50-5935-01	9.9359	653	DECEASED
TCGA-44-7672-01	10.2697	719	LIVING
TCGA-44-6774-01	10.7375	658	LIVING
TCGA-97-7553-01	10.4123	1870	LIVING
TCGA-44-6148-01	10.4409	704	LIVING
TCGA-86-7714-01	10.1632	625	DECEASED
TCGA-44-2661-01	9.7086	1159	LIVING
TCGA-91-6828-01	10.3553	323	LIVING
TCGA-38-6178-01	10.8054	448	LIVING
TCGA-05-4426-01	9.5751	791	LIVING
TCGA-93-7348-01	10.3252	531	LIVING
TCGA-97-8552-01	10.1315	626	LIVING
TCGA-44-3398-01	9.9163	1163	LIVING
TCGA-97-7554-01	10.4483	775	LIVING
TCGA-86-8279-01	10.825	949	LIVING
TCGA-91-8497-01	9.843	434	DECEASED
TCGA-83-5908-01	10.0204	824	LIVING
TCGA-97-8547-01	10.3831	657	LIVING
TCGA-50-5055-01	10.3726	1830	DECEASED

TCGA-97-8175-01	10.5652	551	LIVING
TCGA-86-8668-01	10.4058	423	LIVING
TCGA-91-8496-01	10.5079	505	LIVING
TCGA-86-8278-01	10.7799	944	LIVING
TCGA-55-1594-01	10.5475	1178	LIVING
TCGA-50-5049-01	10.8049	3094	LIVING
TCGA-97-7552-01	10.788	1932	LIVING
TCGA-73-7499-01	10.3991	1531	DECEASED
TCGA-97-8177-01	10.3831	499	LIVING
TCGA-86-8280-01	10.7516	701	LIVING
TCGA-86-7953-01	10.4144	997	LIVING
TCGA-49-AARQ-01	9.9145	6732	LIVING
TCGA-97-A4M6-01	10.0715	568	LIVING
TCGA-NJ-A7XG-01	10.6632	617	LIVING
TCGA-93-A4JQ-01	10.7881	526	LIVING
TCGA-49-AAR4-01	10.0459	879	DECEASED
TCGA-49-AARE-01	10.5034	1229	DECEASED
TCGA-97-A4M1-01	10.2565	601	LIVING
TCGA-MP-A4SW-01	10.5991	1778	DECEASED
TCGA-MP-A4T9-01	9.9388	1265	DECEASED
TCGA-44-2665-01	10.7543	1301	LIVING
TCGA-L9-A743-01	9.9686	664	LIVING
TCGA-86-A4P8-01	9.8474	805	LIVING
TCGA-91-A4BC-01	10.5155	44	LIVING
TCGA-55-A48Z-01	10.3415	651	LIVING
TCGA-MP-A4TJ-01	10.0143	339	DECEASED
TCGA-55-A48X-01	9.9616	689	LIVING
TCGA-86-A4P7-01	10.0878	415	LIVING
TCGA-MN-A4N5-01	10.0994	84	LIVING
TCGA-53-A4EZ-01	10.2047	1071	LIVING
TCGA-49-AAQV-01	10.5385	677	DECEASED
TCGA-49-AAR2-01	8.8515	2224	LIVING
TCGA-93-A4JP-01	10.1209	578	LIVING
TCGA-62-A46P-01	7.9563	594	DECEASED
TCGA-97-A4M2-01	10.7063	624	LIVING
TCGA-L4-A4E6-01	10.3866	435	LIVING
TCGA-S2-AA1A-01	9.49	513	LIVING
TCGA-49-AARR-01	10.6329	4992	LIVING
TCGA-L9-A7SV-01	10.3148	565	LIVING
TCGA-97-A4M3-01	10.513	540	LIVING
TCGA-49-AAR3-01	10.7925	1893	LIVING
TCGA-99-AA5R-01	10.0611	658	LIVING
TCGA-MP-A4TH-01	10.0717	741	LIVING
TCGA-62-A46Y-01	10.5178	414	DECEASED
TCGA-93-A4JN-01	9.4211	718	LIVING
TCGA-55-A4DG-01	10.2056	608	LIVING
TCGA-44-6775-01	9.9939	705	LIVING
TCGA-62-A46S-01	8.4738	1653	DECEASED
TCGA-44-A479-01	8.598	486	LIVING
TCGA-44-A47G-01	10.0249	351	LIVING
TCGA-97-A4M7-01	9.4935	629	LIVING
TCGA-O1-A52J-01	10.1313	1798	DECEASED
TCGA-95-A4VK-01	10.4635	651	LIVING
TCGA-MP-A5C7-01	10.3026	2248	LIVING
TCGA-MP-A4T6-01	10.3116	1790	DECEASED
TCGA-NJ-A55A-01	10.5677	15	LIVING
TCGA-53-7626-01	10.5781	929	DECEASED
TCGA-67-3771-01	9.9	610	LIVING
TCGA-J2-A4AE-01	10.1614	1079	LIVING
TCGA-MP-A4TC-01	10.788	74	DECEASED
TCGA-62-A46V-01	10.5652	2199	LIVING
TCGA-44-A47B-01	9.356	287	LIVING
TCGA-55-A57B-01	10.2954	546	LIVING
TCGA-97-A4LX-01	10.723	614	LIVING
TCGA-97-A4M5-01	10.617	634	LIVING
TCGA-62-A470-01	9.2628	1194	DECEASED

Table S2

Positive regulation of I-kappaB kinase/NF-kappaB signaling and NF-kappaB transcription factor activity

ID	gene_name	gene_chr	gene_strand	gene_biotype	NCD_1_fpkm	NCD_2_fpkm	NCD_3_fpkm	D52_1_fpkm	D52_2_fpkm	D52_3_fpkm	D52vsNCD_log2FoldChange	D52vsNCD_FoldChange	D52vsNCD_pvalue	D52vsNCD_padj
ENSG00000167191	GPRC5B	16	-	protein_coding	5.317987505	4.959059636	8.129506027	2.166708547	1.872813864	2.289767667	-1.540607028	0.343740792	3.75E-12	2.27E-10
ENSG00000184304	PRKD1	14	-	protein_coding	2.186765711	2.373484399	2.438120409	1.076385029	0.828819951	0.779524873	-1.384593092	0.382997507	1.06E-11	5.91E-10
ENSG00000181104	F2R	5	+	protein_coding	6.035488785	5.584950427	6.605711275	2.81167865	3.034067019	3.876288905	-0.906659092	0.533418922	6.64E-07	1.43E-05
ENSG00000097007	ABL1	9	+	protein_coding	41.76164488	40.09235279	43.98592983	29.32191617	28.13879906	28.43304199	-0.551031162	0.682532116	1.01E-06	2.08E-05
ENSG00000119401	TRIM32	9	+	protein_coding	17.24003451	16.75659484	17.46107016	11.91009593	11.25457414	10.1183124	-0.628814417	0.646707651	1.68E-06	3.27E-05
ENSG00000172936	MYD88	3	+	protein_coding	9.585662466	9.378872	9.108469049	6.309539317	6.353956857	5.937013079	-0.593884192	0.662556691	4.67E-06	8.22E-05
ENSG00000137752	CASP1	11	-	protein_coding	0.906676439	0.741122224	1.08566025	0.191719562	0.171854385	0.457443119	-1.738059468	0.299772622	2.58E-05	0.000370532
ENSG00000198121	LPAR1	9	-	protein_coding	0.274443179	0.219657769	0.598499254	0	0.026965653	0.099204467	-3.106937787	0.116069612	6.33E-05	0.000806103
ENSG00000100614	PPM1A	14	+	protein_coding	9.497708145	9.569859989	9.24480713	6.702720366	6.7233353	7.201215473	-0.456877652	0.72856134	7.13E-05	0.000892297
ENSG00000121858	TNFSF10	3	-	protein_coding	0.092433439	0.225467482	0.508637923	0	0	0.047519882	-4.126686525	0.057245793	0.000159918	0.001772026
ENSG00000116729	WLS	1	-	protein_coding	13.64027391	13.85194439	12.95435367	9.796251843	10.18862406	8.161351069	-0.522903786	0.695969609	0.000298111	0.002973226
ENSG00000129353	SLC44A2	19	+	protein_coding	13.15022201	11.69301818	17.07201576	7.339889323	7.919600956	10.27523393	-0.715024466	0.609194801	0.000398782	0.003779429
ENSG00000132256	TRIM5	11	-	protein_coding	6.764049258	6.474693834	7.671857482	4.215281213	4.816884739	5.230394462	-0.551632496	0.682247687	0.00048633	0.004464907
ENSG00000102471	NDFIP2	13	+	protein_coding	5.992455342	5.853787176	6.0133143	4.152509805	4.70099414	4.172179546	-0.454726307	0.729648581	0.001059542	0.008493234
ENSG00000112343	TRIM38	6	+	protein_coding	2.652536476	2.430937982	2.844845067	1.562871878	1.838787473	2.060107811	-0.537295987	0.689061192	0.00150752	0.011427598
ENSG00000128284	APOL3	22	-	protein_coding	1.021533171	1.039464572	1.306280543	0.548164109	0.658849664	0.783091586	-0.757046316	0.591706514	0.001563072	0.011771448
ENSG00000003400	CASP10	2	+	protein_coding	1.41774798	1.540171861	1.785986842	1.026423839	1.0725951	1.091482854	-0.571278868	0.67301993	0.001581386	0.011899672
ENSG00000145632	PLK2	5	-	protein_coding	21.84429274	21.05793358	20.99166446	17.42522429	16.64532777	15.68851029	-0.360874062	0.778692663	0.002342082	0.016364467
ENSG00000174130	TLR6	4	-	protein_coding	1.737646538	1.946268186	1.742949728	0.844372183	1.108802919	1.43569494	-0.677564275	0.625219953	0.003184209	0.020934675
ENSG00000019582	CD74	5	-	protein_coding	2.307732916	2.500312111	3.480141216	1.739603631	1.620259705	2.034448698	-0.619599694	0.650851495	0.008308503	0.044989583
ENSG00000136869	TLR4	9	+	protein_coding	2.805792235	2.828724269	3.049193625	0.594828027	0.68446068	0.705682965	-2.128243466	0.228736188	5.33E-44	4.29E-41
ENSG00000114251	WNT5A	3	-	protein_coding	0.694381684	0.729259777	1.118078885	0.06589469	0.040430859	0.078089486	-3.795723726	0.072006765	1.33E-22	2.74E-20
ENSG00000164342	TLR3	4	+	protein_coding	1.230698478	1.242194831	1.152366032	0.369040197	0.472574001	0.368166014	-1.579275959	0.334649796	1.37E-12	8.73E-11
ENSG00000120885	CLU	8	-	protein_coding	8.626127429	8.672923044	14.23010795	3.589902427	3.384620105	3.953792842	-1.528700853	0.34658933	5.39E-12	3.22E-10
ENSG00000104899	AMH	19	+	protein_coding	13.74643595	15.55469493	12.10483901	6.474537083	7.051832651	4.194492955	-1.223808376	0.428151007	2.87E-08	8.38E-07
ENSG00000120337	TNFSF18	1	-	protein_coding	0.746065513	0.953246374	1.11965742	0.044134645	0.037234446	0.287663312	-2.93710152	0.130570282	4.96E-06	8.69E-05
ENSG00000167632	TRAPPC9	8	-	protein_coding	0.903433686	0.921953181	1.128934019	0.650489826	0.699513324	0.652604994	-0.559918307	0.678340574	0.003764366	0.023964602

Negative regulation of I-kappaB kinase/NF-kappaB signaling and NF-kappaB transcription factor activity

ID	gene_name	gene_chr	gene_strand	gene_biotype	NCD_1_fpkm	NCD_2_fpkm	NCD_3_fpkm	D52_1_fpkm	D52_2_fpkm	D52_3_fpkm	D52vsNCD_log2FoldChange	D52vsNCD_FoldChange	D52vsNCD_pvalue	D52vsNCD_padj
ENSG00000136848	DAB2IP	9	+	protein_coding	6.90948365	6.372642427	7.196413222	14.19589143	13.7622623	13.93688202	1.032425566	2.045460342	9.16E-18	1.11E-15
ENSG00000101782	RIOK3	18	+	protein_coding	10.67389225	11.20276249	11.62925384	17.10811491	18.95871583	17.8453527	0.686517875	1.609394352	2.34E-08	6.98E-07
ENSG00000145901	TNIP1	5	-	protein_coding	40.84113963	40.07835319	36.00334627	56.65681643	55.63303085	52.35558828	0.493774953	1.408124555	4.50E-05	0.000598737
ENSG00000123240	OPTN	10	+	protein_coding	16.45302014	16.76437181	20.49580756	24.24265032	26.24403731	35.95920974	0.686567504	1.609449717	0.000323308	0.00316979
ENSG00000091592	NLRP1	17	-	protein_coding	1.033198993	1.16840476	1.504598978	1.688308685	1.926376718	3.95904751	1.031322717	2.043897314	0.00087837	0.007256667
ENSG00000165233	CARD19	9	+	protein_coding	5.939050938	5.930260702	11.44099146	13.31780464	14.77863061	13.88696074	0.849047921	1.801311792	0.000979491	0.007951019
ENSG00000137802	MAPKBP1	15	+	protein_coding	1.684093758	1.646524831	1.810271642	2.276098264	2.270362858	2.414119686	0.437057255	1.353840007	0.001976017	0.014276301
ENSG00000101997	CCDC22	X	+	protein_coding	12.19211914	11.0139979	9.797574587	20.20068668	16.37380303	12.52860945	0.572632751	1.487235127	0.005121947	0.030521924
ENSG00000141480	ARRB2	17	+	protein_coding	4.715622905	4.616937773	5.647494368	11.00684938	11.33814417	10.26759883	1.122472248	2.177197445	5.51E-14	4.43E-12
ENSG00000083799	CYLD	16	+	protein_coding	6.283521866	5.658641839	5.832793085	8.79450774	9.026958706	9.408637752	0.615133243	1.531699454	3.07E-07	7.06E-06
ENSG00000106052	TAX1BP1	7	+	protein_coding	9.239311909	9.227413552	8.235428936	12.84129884	14.3700665	14.85599074	0.655828155	1.575520094	6.77E-07	1.45E-05
ENSG00000204498	NFKBIL1	6	+	protein_coding	9.122772231	8.094093922	10.17797636	15.24364498	13.8900598	13.75304598	0.645705628	1.564504305	4.84E-05	0.000637755
ENSG00000134070	IRAK2	3	+	protein_coding	21.72093807	19.88409287	17.08266299	27.11086203	25.02958655	31.58106854	0.512289686	1.426312085	0.001416035	0.010849808
ENSG00000149600	COMMD7	20	-	protein_coding	3.616597397	3.636448452	3.029723155	4.647186834	5.049071511	4.981753496	0.513800821	1.427806843	0.003084552	0.020421076

Table S3

Gene Name	BioID		log2 (Fold Change)	z score	p value	Signature p value
	FKBP4	CON				
FLNA	26.026	0	5.702	5.995	2.03E-09	TRUE
FKBP4	22.937	0	5.520	5.642	1.68E-08	TRUE
BTF3L4	20.438	0	5.353	5.333	9.63E-08	TRUE
ANXA2	19.524	0	5.287	5.215	1.84E-07	TRUE
SIM2	19.434	0	5.280	5.203	1.96E-07	TRUE
HSP90AA1	19.338	0	5.273	5.190	2.10E-07	TRUE
MYOF	18.379	0	5.200	5.061	4.17E-07	TRUE
SHROOM3	17.951	0	5.166	5.002	5.67E-07	TRUE
HSP90AB1	17.853	0	5.158	4.988	6.09E-07	TRUE
HSP90AB3P	17.853	0	5.158	4.988	6.09E-07	TRUE
PRRC2C	30.721	0	5.941	4.889	1.01E-06	TRUE
IGKV3D-11	16.787	0	5.069	4.837	1.32E-06	TRUE
IGKV3-11	16.787	0	5.069	4.837	1.32E-06	TRUE
DNAJC11	16.692	0	5.061	4.823	1.41E-06	TRUE
ALG10	29.698	0	5.892	4.799	1.59E-06	TRUE
ALG10B	29.698	0	5.892	4.799	1.59E-06	TRUE
TJP2	29.648	0	5.890	4.795	1.63E-06	TRUE
KCNK10	28.550	0	5.835	4.696	2.65E-06	TRUE
XPO5	28.498	0	5.833	4.691	2.72E-06	TRUE
VPS45	28.403	0	5.828	4.683	2.83E-06	TRUE
PLEC	15.385	0	4.943	4.628	3.68E-06	TRUE
DHX29	15.370	0	4.942	4.626	3.72E-06	TRUE
DNM1L	27.686	0	5.791	4.617	3.90E-06	TRUE
VPS33B	27.478	0	5.780	4.597	4.28E-06	TRUE
DVL1	27.439	0	5.778	4.594	4.35E-06	TRUE
DVL3	27.383	0	5.775	4.588	4.46E-06	TRUE
EIF2A	27.383	0	5.775	4.588	4.46E-06	TRUE
ARHGEF16	26.976	0	5.754	4.550	5.36E-06	TRUE
PRRC2A	26.826	0	5.746	4.536	5.73E-06	TRUE
XRN1	26.726	0	5.740	4.527	5.99E-06	TRUE
CORO1B	26.726	0	5.740	4.527	5.99E-06	TRUE
POLD1	26.646	0	5.736	4.519	6.21E-06	TRUE
SRGAP1	26.604	0	5.734	4.515	6.33E-06	TRUE
VCPIP1	26.384	0	5.722	4.494	6.98E-06	TRUE
NT5C2	26.287	0	5.716	4.485	7.29E-06	TRUE
VIPAS39	26.239	0	5.714	4.480	7.45E-06	TRUE
LUC7L	26.235	0	5.713	4.480	7.46E-06	TRUE
AFDN	26.042	0	5.703	4.462	8.14E-06	TRUE
SF3B3	26.030	0	5.702	4.460	8.18E-06	TRUE
NONO	26.019	0	5.701	4.459	8.22E-06	TRUE
SYNJ2	25.812	0	5.690	4.439	9.03E-06	TRUE
RBSN	25.792	0	5.689	4.437	9.11E-06	TRUE
DIP2B	25.741	0	5.686	4.433	9.31E-06	TRUE
EIF2S1	25.736	0	5.686	4.432	9.34E-06	TRUE
DPP9	25.612	0	5.679	4.420	9.87E-06	TRUE
GRK2	25.573	0	5.677	4.416	1.00E-05	TRUE
RFC4	25.526	0	5.674	4.412	1.03E-05	TRUE
PIK3CA	25.414	0	5.668	4.401	1.08E-05	TRUE
PSPC1	25.404	0	5.667	4.400	1.08E-05	TRUE
ATAD3B	25.371	0	5.665	4.396	1.10E-05	TRUE
SLFN5	25.257	0	5.659	4.385	1.16E-05	TRUE
CD2AP	25.133	0	5.652	4.373	1.22E-05	TRUE
MAPRE1	25.069	0	5.648	4.367	1.26E-05	TRUE
GMPS	25.053	0	5.647	4.365	1.27E-05	TRUE
OCRL	25.002	0	5.644	4.360	1.30E-05	TRUE
POTEF	24.993	0	5.643	4.359	1.30E-05	TRUE
TCF25	24.989	0	5.643	4.359	1.31E-05	TRUE
EFCAB7	24.941	0	5.640	4.354	1.34E-05	TRUE
CAPN7	24.941	0	5.640	4.354	1.34E-05	TRUE
VPS11	24.923	0	5.639	4.352	1.35E-05	TRUE
ABR	24.918	0	5.639	4.352	1.35E-05	TRUE
RAB11FIP2	24.876	0	5.637	4.348	1.37E-05	TRUE
KIAA1211L	24.867	0	5.636	4.347	1.38E-05	TRUE
LRRC31	24.775	0	5.631	4.338	1.44E-05	TRUE
NAA35	24.729	0	5.628	4.333	1.47E-05	TRUE
TRIP10	24.723	0	5.628	4.333	1.47E-05	TRUE
RECQL	24.702	0	5.627	4.331	1.49E-05	TRUE
GNPDA1	24.692	0	5.626	4.330	1.49E-05	TRUE
VIPR1	24.676	0	5.625	4.328	1.51E-05	TRUE
PDS5A	24.665	0	5.624	4.327	1.51E-05	TRUE
GLB1L	24.654	0	5.624	4.326	1.52E-05	TRUE
CTPS2	24.638	0	5.623	4.324	1.53E-05	TRUE
SNX2	24.632	0	5.622	4.324	1.54E-05	TRUE
MYH10	24.626	0	5.622	4.323	1.54E-05	TRUE
FER1L6	24.593	0	5.620	4.320	1.56E-05	TRUE

SEC11B	24.564	0	5.618	4.317	1.58E-05	TRUE
SRP68	24.564	0	5.618	4.317	1.58E-05	TRUE
TP53	24.558	0	5.618	4.316	1.59E-05	TRUE
SNX18	24.517	0	5.616	4.312	1.62E-05	TRUE
ACTR2	24.511	0	5.615	4.311	1.62E-05	TRUE
STXBP3	24.468	0	5.613	4.307	1.65E-05	TRUE
LRRC47	24.468	0	5.613	4.307	1.65E-05	TRUE
EML4	24.468	0	5.613	4.307	1.65E-05	TRUE
ILK	24.461	0	5.612	4.307	1.66E-05	TRUE
CACYBP	24.384	0	5.608	4.299	1.72E-05	TRUE
FAM91A1	24.331	0	5.605	4.293	1.76E-05	TRUE
CWF19L2	24.303	0	5.603	4.291	1.78E-05	TRUE
PLCB3	24.296	0	5.603	4.290	1.79E-05	TRUE
EIF3J	24.282	0	5.602	4.289	1.80E-05	TRUE
PSMD5	24.261	0	5.601	4.286	1.82E-05	TRUE
COPB2	24.217	0	5.598	4.282	1.85E-05	TRUE
RPS6KA1	24.187	0	5.596	4.279	1.88E-05	TRUE
CAMSAP3	24.187	0	5.596	4.279	1.88E-05	TRUE
TUBA3E	24.179	0	5.596	4.278	1.88E-05	TRUE
ZC3HAV1	24.179	0	5.596	4.278	1.88E-05	TRUE
PIK3C2A	24.164	0	5.595	4.277	1.90E-05	TRUE
ADAM20	24.164	0	5.595	4.277	1.90E-05	TRUE
RANGAP1	24.157	0	5.594	4.276	1.90E-05	TRUE
EHD1	24.149	0	5.594	4.275	1.91E-05	TRUE
BCR	24.141	0	5.593	4.274	1.92E-05	TRUE
RPAP3	24.141	0	5.593	4.274	1.92E-05	TRUE
DGKA	24.117	0	5.592	4.272	1.94E-05	TRUE
MYH16	24.109	0	5.592	4.271	1.94E-05	TRUE
DBNL	24.101	0	5.591	4.270	1.95E-05	TRUE
PIK3C2B	24.085	0	5.590	4.269	1.97E-05	TRUE
TBC1D2	24.069	0	5.589	4.267	1.98E-05	TRUE
VPS33A	24.019	0	5.586	4.262	2.02E-05	TRUE
MICALL2	24.002	0	5.585	4.260	2.04E-05	TRUE
OGT	23.993	0	5.585	4.260	2.05E-05	TRUE
MAP3K7	23.993	0	5.585	4.260	2.05E-05	TRUE
RAPH1	23.985	0	5.584	4.259	2.06E-05	TRUE
UBASH3B	23.985	0	5.584	4.259	2.06E-05	TRUE
ORC3	23.976	0	5.584	4.258	2.06E-05	TRUE
WDR1	23.949	0	5.582	4.255	2.09E-05	TRUE
SKIV2L	23.923	0	5.580	4.252	2.12E-05	TRUE
BCAS3	23.913	0	5.580	4.251	2.12E-05	TRUE
FAM83H	23.886	0	5.578	4.249	2.15E-05	TRUE
STAT3	23.876	0	5.578	4.248	2.16E-05	TRUE
CAPN2	23.848	0	5.576	4.245	2.19E-05	TRUE
PRKAR2A	23.829	0	5.575	4.243	2.21E-05	TRUE
WASHC5	23.809	0	5.573	4.241	2.23E-05	TRUE
NUDC	23.809	0	5.573	4.241	2.23E-05	TRUE
RNPC3	23.770	0	5.571	4.237	2.27E-05	TRUE
AGPS	23.729	0	5.569	4.233	2.31E-05	TRUE
IGF2BP3	23.729	0	5.569	4.233	2.31E-05	TRUE
CSDE1	23.729	0	5.569	4.233	2.31E-05	TRUE
ARFGAP2	23.718	0	5.568	4.232	2.32E-05	TRUE
NSFL1C	23.708	0	5.567	4.231	2.33E-05	TRUE
RIPK2	23.697	0	5.567	4.229	2.34E-05	TRUE
LIN54	23.686	0	5.566	4.228	2.35E-05	TRUE
SHMT2	23.665	0	5.565	4.226	2.38E-05	TRUE
RPTOR	23.665	0	5.565	4.226	2.38E-05	TRUE
GEMIN5	23.665	0	5.565	4.226	2.38E-05	TRUE
SNX8	23.654	0	5.564	4.225	2.39E-05	TRUE
DVL2	23.621	0	5.562	4.222	2.42E-05	TRUE
VLDLR	23.610	0	5.561	4.221	2.44E-05	TRUE
AGO3	23.598	0	5.561	4.219	2.45E-05	TRUE
SMARCAL1	23.598	0	5.561	4.219	2.45E-05	TRUE
GSK3B	23.587	0	5.560	4.218	2.46E-05	TRUE
MIOS	23.587	0	5.560	4.218	2.46E-05	TRUE
RRM1	23.552	0	5.558	4.215	2.50E-05	TRUE
CTPS1	23.540	0	5.557	4.213	2.51E-05	TRUE
SPIRE1	23.529	0	5.556	4.212	2.53E-05	TRUE
TAB3	23.504	0	5.555	4.210	2.56E-05	TRUE
MPHOSPH8	23.504	0	5.555	4.210	2.56E-05	TRUE
TAB1	23.480	0	5.553	4.207	2.58E-05	TRUE
EEF1B2	23.455	0	5.552	4.205	2.61E-05	TRUE
DPYSL2	23.455	0	5.552	4.205	2.61E-05	TRUE
LRRC40	23.455	0	5.552	4.205	2.61E-05	TRUE
OAS3	23.455	0	5.552	4.205	2.61E-05	TRUE
SRP72	23.430	0	5.550	4.202	2.64E-05	TRUE
RAB3GAP2	23.430	0	5.550	4.202	2.64E-05	TRUE
AGO4	23.430	0	5.550	4.202	2.64E-05	TRUE
AGO1	23.430	0	5.550	4.202	2.64E-05	TRUE
G3BP2	23.430	0	5.550	4.202	2.64E-05	TRUE
MTMR12	23.404	0	5.549	4.199	2.68E-05	TRUE

AGO2	23.404	0	5.549	4.199	2.68E-05	TRUE
SH3KBP1	23.378	0	5.547	4.197	2.71E-05	TRUE
VPS53	23.324	0	5.544	4.191	2.77E-05	TRUE
FBXL18	23.324	0	5.544	4.191	2.77E-05	TRUE
NT5DC2	23.310	0	5.543	4.190	2.79E-05	TRUE
COBL	23.296	0	5.542	4.188	2.81E-05	TRUE
MINK1	23.296	0	5.542	4.188	2.81E-05	TRUE
STXBP1	23.282	0	5.541	4.187	2.83E-05	TRUE
ABCB1	23.268	0	5.540	4.185	2.85E-05	TRUE
ABCB4	23.268	0	5.540	4.185	2.85E-05	TRUE
HPS3	23.268	0	5.540	4.185	2.85E-05	TRUE
SARS	23.249	0	5.539	4.184	2.87E-05	TRUE
PITPNM1	23.240	0	5.539	4.183	2.88E-05	TRUE
RAPGEF6	23.238	0	5.538	4.182	2.89E-05	TRUE
ARHGEF1	23.213	0	5.537	4.180	2.92E-05	TRUE
DHX38	23.205	0	5.536	4.179	2.93E-05	TRUE
AKAP2	23.192	0	5.536	4.178	2.95E-05	TRUE
SH3PXD2B	23.190	0	5.535	4.177	2.95E-05	TRUE
PACS1	23.170	0	5.534	4.175	2.97E-05	TRUE
MTM1	23.169	0	5.534	4.175	2.98E-05	TRUE
AFAP1L2	23.152	0	5.533	4.174	3.00E-05	TRUE
SLFN13	23.111	0	5.531	4.169	3.06E-05	TRUE
INPPL1	23.100	0	5.530	4.168	3.07E-05	TRUE
SCFD1	23.074	0	5.528	4.165	3.11E-05	TRUE
ATXN2L	23.071	0	5.528	4.165	3.11E-05	TRUE
AGPAT1	23.071	0	5.528	4.165	3.11E-05	TRUE
TEX9	23.048	0	5.527	4.163	3.14E-05	TRUE
ANLN	23.044	0	5.526	4.162	3.15E-05	TRUE
ARHGAP29	23.041	0	5.526	4.162	3.15E-05	TRUE
SETMAR	23.021	0	5.525	4.160	3.18E-05	TRUE
SH3GL1	23.017	0	5.525	4.160	3.19E-05	TRUE
TBL3	23.007	0	5.524	4.159	3.20E-05	TRUE
FAM83F	22.981	0	5.522	4.156	3.24E-05	TRUE
MYL6B	22.976	0	5.522	4.155	3.25E-05	TRUE
MYL6	22.976	0	5.522	4.155	3.25E-05	TRUE
EGLN1	22.967	0	5.522	4.154	3.26E-05	TRUE
BAIAP2	22.955	0	5.521	4.153	3.28E-05	TRUE
CPNE2	22.948	0	5.520	4.152	3.29E-05	TRUE
OSBPL10	22.941	0	5.520	4.152	3.30E-05	TRUE
PPP2R2B	22.937	0	5.520	4.151	3.31E-05	TRUE
NACA2	22.937	0	5.520	4.151	3.31E-05	TRUE
ABCF3	22.930	0	5.519	4.150	3.32E-05	TRUE
TCAF2	22.923	0	5.519	4.150	3.33E-05	TRUE
PYGB	22.917	0	5.518	4.149	3.34E-05	TRUE
FAM129B	22.913	0	5.518	4.149	3.34E-05	TRUE
CARD11	22.912	0	5.518	4.149	3.35E-05	TRUE
MAP4K2	22.904	0	5.518	4.148	3.36E-05	TRUE
PTPN23	22.873	0	5.516	4.145	3.40E-05	TRUE
RASAL2	22.823	0	5.512	4.139	3.48E-05	TRUE
ANXA2P2	22.819	0	5.512	4.139	3.49E-05	TRUE
NDUFS2	22.807	0	5.511	4.138	3.51E-05	TRUE
ACSL4	22.797	0	5.511	4.137	3.52E-05	TRUE
CAMK2D	22.794	0	5.511	4.136	3.53E-05	TRUE
HPS6	22.782	0	5.510	4.135	3.55E-05	TRUE
PLS3	22.772	0	5.509	4.134	3.56E-05	TRUE
HSP90AA2P	22.749	0	5.508	4.132	3.60E-05	TRUE
FNBP1L	22.747	0	5.508	4.131	3.60E-05	TRUE
MYO1C	22.731	0	5.507	4.130	3.63E-05	TRUE
GNE	22.722	0	5.506	4.129	3.65E-05	TRUE
SRP54	22.710	0	5.505	4.128	3.67E-05	TRUE
PA2G4	22.703	0	5.505	4.127	3.68E-05	TRUE
DZIP1	22.682	0	5.503	4.125	3.71E-05	TRUE
PDLIM1	22.650	0	5.501	4.121	3.77E-05	TRUE
WDR70	22.650	0	5.501	4.121	3.77E-05	TRUE
DHX57	22.645	0	5.501	4.121	3.78E-05	TRUE
MTREX	22.639	0	5.501	4.120	3.79E-05	TRUE
TBK1	22.634	0	5.500	4.120	3.79E-05	TRUE
DIEXF	22.619	0	5.499	4.118	3.82E-05	TRUE
HSPA1L	22.610	0	5.499	4.117	3.84E-05	TRUE
CKAP5	22.610	0	5.499	4.117	3.84E-05	TRUE
UNC45A	22.589	0	5.498	4.115	3.87E-05	TRUE
SLC27A4	22.582	0	5.497	4.114	3.89E-05	TRUE
DAB2IP	22.573	0	5.497	4.113	3.90E-05	TRUE
SACM1L	22.573	0	5.497	4.113	3.90E-05	TRUE
DNAJA2	22.543	0	5.495	4.110	3.96E-05	TRUE
SUPV3L1	22.533	0	5.494	4.109	3.97E-05	TRUE
CAMK2G	22.526	0	5.494	4.108	3.99E-05	TRUE
PPP1R13L	22.517	0	5.493	4.107	4.00E-05	TRUE
POLD2	22.490	0	5.491	4.104	4.05E-05	TRUE
SMG9	22.485	0	5.491	4.104	4.06E-05	TRUE
CLEC16A	22.480	0	5.491	4.103	4.07E-05	TRUE

DOCK9	22.480	0	5.491	4.103	4.07E-05	TRUE
PIK3CB	22.465	0	5.490	4.102	4.10E-05	TRUE
CLINT1	22.460	0	5.489	4.101	4.11E-05	TRUE
